# YY1 modulates the radiosensitivity of esophageal squamous cell carcinoma through KIF3B-mediated Hippo signaling pathway

**DOI:** 10.1038/s41419-023-06321-x

**Published:** 2023-12-08

**Authors:** Chunyan Zheng, Zhe Li, Chuanxi Zhao, Xiaoyang Yin, Lei Feng, Zhongtang Wang, Chengxin Liu, Baosheng Li

**Affiliations:** 1 Shandong First Medical University and Shandong Academy of Medical Sciences, Jinan, China; 2 Department of Radiation Oncology, Shandong Cancer Hospital and Institute, Shandong First Medical University and Shandong Academy of Medical Sciences, Jinan, China; 3 National Key Laboratory of Advanced Drug Delivery and Release Systems, Jinan, China; 4 Department of Pulmonary and Critical Care Medicine, Shandong Provincial Hospital Affiliated to Shandong First Medical University, Jinan, China; 5 Department of Clinical Laboratory, Shandong Cancer Hospital and Institute, Shandong First Medical University and Shandong Academy of Medical Sciences, Jinan, China; 6 Department of Radiation Oncology, The Affiliated Hospital of Qingdao University, Qingdao, China; 7 Department of Radiation Oncology, Shandong Provincial Hospital Affiliated to Shandong First Medical University, Jinan, China

**Keywords:** Cancer therapeutic resistance, Radiotherapy

## Abstract

Radiotherapy is an important strategy in the comprehensive treatment of esophageal squamous cell carcinoma (ESCC). However, effectiveness of radiotherapy is still restricted by radioresistance. Herein, we aimed to understand the mechanisms underlying ESCC radioresistance, for which we looked into the potential role of YY1. YY1 was upregulated in radioresistant tissues and correlated with poor prognosis of patients with ESCC. YY1 depletion enhanced the radiosensitivity of ESCC in vitro and in vivo. Multi-group sequencing showed that downregulation of YY1 inhibited the transcriptional activity of Kinesin Family Member 3B (KIF3B), which further activated the Hippo signaling pathway by interacting with Integrin-beta1 (ITGB1). Once the Hippo pathway was activated, its main effector, Yes-associated protein 1 (YAP1), was phosphorylated in the cytoplasm and its expression reduced in the nucleus, thus enhancing the radiosensitivity by regulating its targeted genes. Our study provides new insights into the mechanisms underlying ESCC radioresistance and highlights the potential role of YY1 as a therapeutic target for ESCC.

## Introduction

Esophageal cancer (EC) is a prevalent malignant tumor and ranks as the sixth leading cause of cancer-related deaths globally [1]. It comprises two primary pathological types, adenocarcinoma and esophageal squamous cell carcinoma (ESCC), ESCC accounts for more than 90% of patients with EC in China [2]. Neoadjuvant chemoradiotherapy (nCRT) plus surgery is considered to be the standard treatment for locally advanced patients with ESCC, especially those who have potential chance for surgical resection [3]. Nevertheless, intrinsic or acquired radioresistance is a major obstacle hampering the effectiveness of radiotherapy (RT) [4]. Hence, new biomarkers need to be identified to clarify the molecular mechanisms underlying radioresistance in ESCC.

The transcriptional factor Yin Yang 1 (YY1), is a zinc-finger protein and belongs to the GLI-Kruppel family [5]. It is a complex protein that takes part in suppressing or stimulating promoters of target genes, which, depending on the interaction partner and the promoter environment, may participate in approximately 10% of transcriptional control in the entire mammalian genome [5–7]. YY1 plays a role in multiple biological functions such as apoptosis and metabolism [8, 9]. Morever, the expression of YY1 is significantly higher in malignant tumors [10, 11]. Reportedly, YY1 is related to therapy resistance, especially chemotherapy and immune resistance [12–14]. Currently, little is known about the latent role of YY1 in ESCC radiosensitivity.

Kinesin superfamily (KIFs) is a class of conservative molecular motor proteins and plays an important role in mitotic progression, especially Kinesin Family Member 3B (KIF3B) [15]. Considering that dysregulation of mitotic progression could lead to abnormal proliferation of tumor cells, the role of KIF3B in tumor evolution has been studied widely. However, the role of KIF3B in ESCC and its response to RT still remain unclear.

The Hippo pathway is an evolutionarily conserved signaling pathway that regulates cell growth, proliferation, and regeneration [16, 17]. There are two main parts in the pathway, one is the central serine/threonine kinase cascade including STE20-like MST1/2, Large Tumor Suppessor LATS1/2 kinases and their corresponding adaptor proteins SAV1, and MOB1, the other is the transcriptional module Yes-associated protein 1 (YAP1). YAP1 is a transcriptional co-activator whose abnormal regulation is implicated in tumor progression and therapy resistance [18–20]. Once the Hippo pathway is activated, it limits tissue growth and cell proliferation by phosphorylating YAP1 in the cytoplasm and inhibiting the expression of YAP1 in the nucleus. When the Hippo pathway is off, YAP1 is dephosphorylated and is translocated into the nucleus, where it binds to the transcriptional enhanced associate domain (TEAD) family to induce the expression of downstream genes participating in various cellular activities including proliferation, and survival [16, 19].

In our research, we first found YY1 correlated with radioresistance and poor prognosis in patients with ESCC, YY1 depletion can strengthen the radiosensitivity of ESCC. Further studies revealed that YY1 depletion can suppress the transcription of KIF3B, which further activates the Hippo signaling pathway by interacting with Integrin-beta1 (ITGB1) and enhances the radiosensitivity of ESCC. Our study may provide new biomarkers and strategy to allievate radioresistence of patients with ESCC.

## Results

### High expression of YY1 correlates with radioresistance and poor prognosis in patients with ESCC

**Fig. 1:**
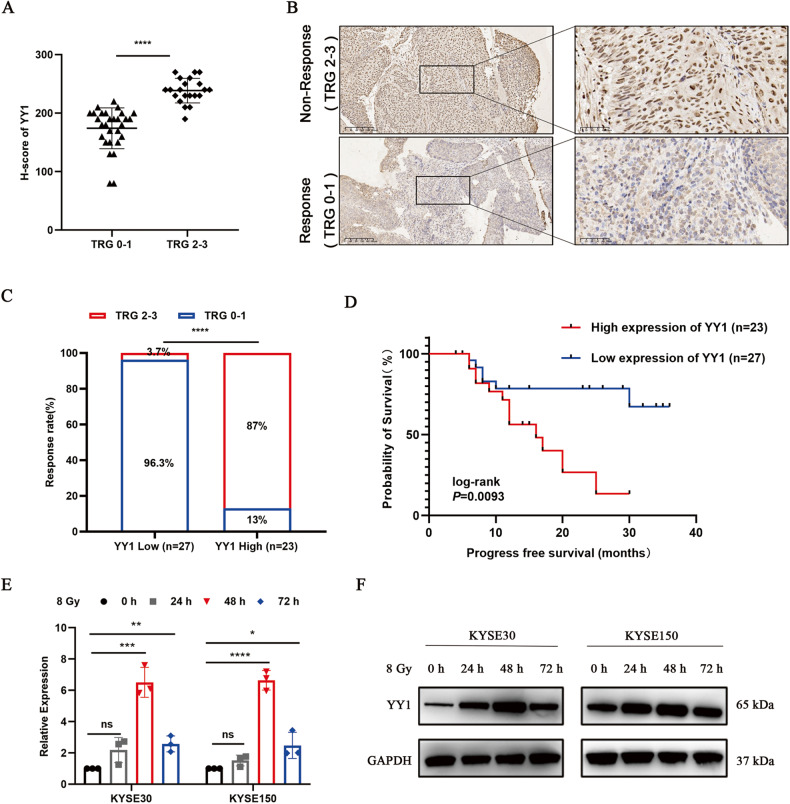
Up-regulated expression of YY1 contributes to poor response to nCRT. **A** H-score of YY1 in TRG 0-1 and TRG 2-3 ESCC tissues from clinical cohorts. **B** Images of IHC staining. Scale bar, 200 μm and 50 μm. **C** Relationship between YY1 expression and response to nCRT. **D** Kaplan-Meier curves displayed the relationship between YY1 expression and progress free survival in ESCC patients received nCRT. **E**, **F** The mRNA and protein expression level of YY1 in ESCC cells treated with 8 Gy irradiation. *P < 0.05, **P < 0.01, ***P < 0.001, ****P < 0.0001, ns: no significance.

To better understand the role of YY1 in ESCC radiosensitivity, we firstly collected 50 endoscopic specimens from patients with ESCC before nCRT, and evaluated the protein expression level of YY1 by IHC staining. Representative images and antibody validation are presented in Fig. S1A, B. TRG system can be regarded as an index to better reveal the prognostic information of nCRT, hence, we divided patients into response (TRG 0-1) and non-response (TRG 2-3) groups based on surgical pathology [21]. We then analyzed the correlation between YY1 expression and TRG. Our results showed that compared to that in the response group, the expression of YY1 was higher in the non-response group, thereby indicating that the lower expression of YY1 correlated with more sensitive to nCRT (Fig. 1A–C). Furthermore, Kaplan-Meier curves indicated that patients with high YY1 expression had shorter survival rates than those with low YY1 expression (Fig. 1D). Interestingly, YY1 mRNA and protein levels were both increased when ESCC cells were exposed to 8 Gy X-ray irradiation (Fig. 1E, F). Collectively, these findings prompted us to wonder whether the expression of YY1 was associated with regulation of radiosensitivity in ESCC.

### YY1 modulates the radiosensitivity of ESCC

**Fig. 2:**
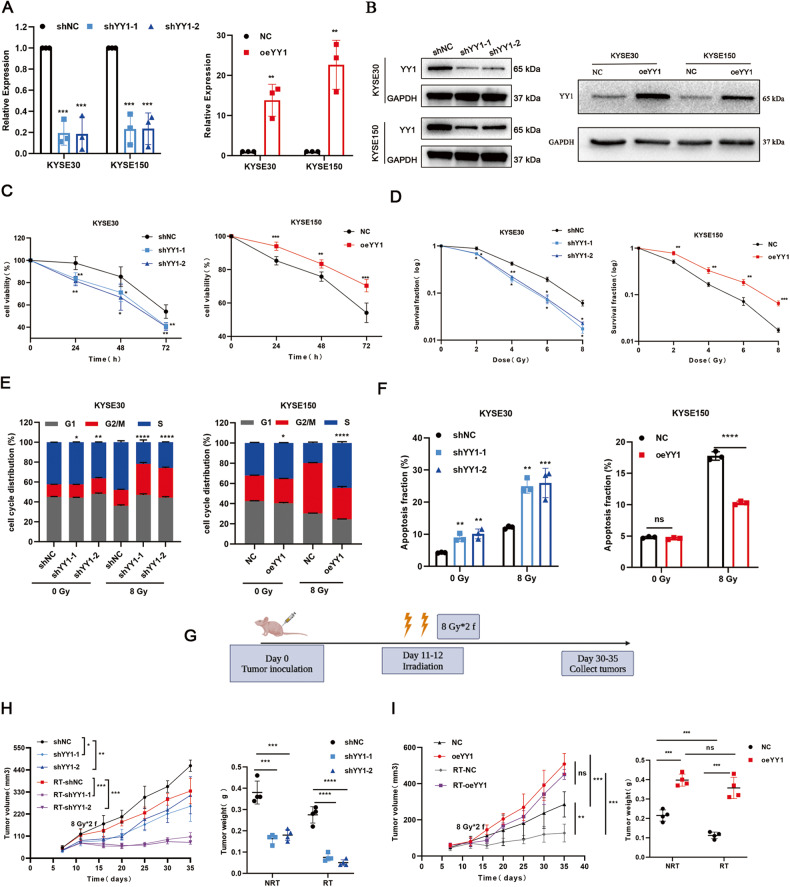
YY1 depletion sensitizes ESCC cells to RT in vitro and in vivo. **A**, **B** Verifying efficiency via RT-qPCR and WB. **C** CCK-8 assay showed the cell viability. **D** Colony formation assays were performed to measure cell colony formation ability after RT. **E**, **F** Flow-cytometry assays were performed to evaluate cell cycle distribution and proportion of apoptosis cells. **G** Schematic representation of tumor inoculation and irradiation in animal model. **H**, **I** Tumor volume and weight of xenografts in different groups. *P < 0.05, **P < 0.01, ***P < 0.001, ****P < 0.0001, ns no significance.

To elucidate the effect of YY1 on ESCC radiosensitivity, lentiviruses were designed to knockdown and upregulate YY1 in ESCC cells (Fig. 2A, B). CCK-8 assay and clonogenic assay displayed that YY1 downregulation decreased cell viability and survival fraction, while its overexpression had the opposite effects (Fig. 2C, D, Fig. S1C–H). We also found that YY1 knockdown increased the G2/M arrest of ESCC cells treated with RT (Fig. 2E, Fig. S1I–K). In addition, apoptosis assays showed that YY1 inhibition facilitated the fraction of radiation-induced apoptotic cells (Fig. 2F, Fig. S2A–D). We observed similar results in animal experiments, in YY1-depletion groups, tumor volume and weight were reduced after X-ray irradiation (Fig. 2G–I and Fig. S2E, F). These findings revealed that YY1 could regulate ESCC radiosensitivity in vitro and in vivo.

### KIF3B is a potential target gene of YY1

**Fig. 3:**
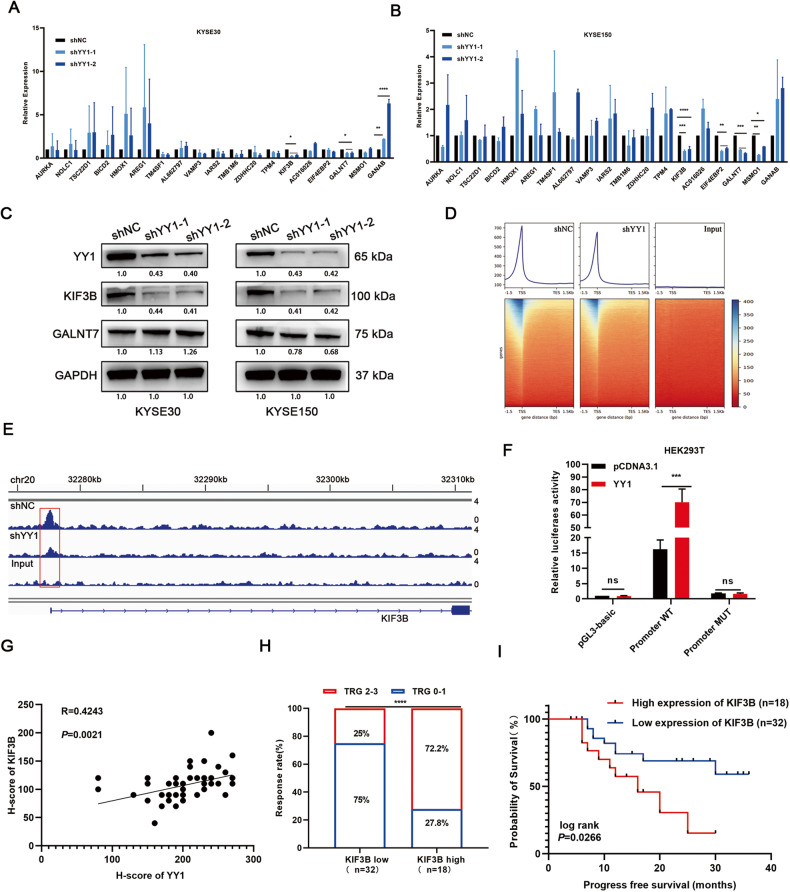
Identifying the target genes of YY1. **A**, **B** RT-qPCR was performed to validate the expression of the top 20 candidate genes. **C** WB showed the protein expression levels of KIF3B and GALNT7 after downregulation YY1. **D**, **E** ChIP-sequencing demonstrated the directly interaction between YY1 and KIF3B promoter. **F** Luciferase assay revealed YY1 could promote the transcriptional activity of KIF3B. **G** Expression of KIF3B was correlated positively with YY1 in endoscopic specimens. **H** Correlation between KIF3B expression and TRG. **I** Kaplan-Meier curves for KIF3B expression and progression free survival in patients with ESCC. *P < 0.05, **P < 0.01, ***P < 0.001, ****P < 0.0001, ns: no significance.

To investigate how depletion of YY1 sensitizes ESCC cells to irradiation, we performed RNA-sequencing to profile the gene expression. We selected the top 20 genes and analyzed the expression changes using RT-qPCR, results showed that after knocking down YY1, expression of KIF3B and GALNT7 decreased in ESCC cells (Fig. S2G, Fig. 3A, B). Among the two candidate target genes, we further measured their protein expression levels, KIF3B decreased significantly in both YY1 depletion cells while GALNT7 protein expression remained inconsistently (Fig. 3C). Moreover, since ChIP-sequencing was regarded as the classical experimental method for studying the interaction between proteins and DNA in vivo, we performed ChIP-sequencing to examine all DNA fragments (Fig. 3D). Notably, a strong enrichment of YY1 signals was detected within the promoter region of KIF3B, which is located on chromosome 20, thereby suggesting that YY1 binds to the specific promoter of KIF3B and influences its transcriptional activity (Fig. 3E). Luciferase assay was performed upon YY1 ectopic expression and the results showed that there was a marked elevation in luciferase activity in the wild type rather than in the mutant type (Fig. 3F), thus confirming the regulatory ability of YY1 on KIF3B. We also found that the expression of KIF3B correlated positively with YY1 in endoscopic specimens (Fig. 3G). Moreover, patients with lower expression of KIF3B correlated with fewer residual tumor and better survival rate, thus indicating YY1 influenced the radiosensitivity of ESCC through regulation of KIF3B (Fig. 3H, I and Fig. S3A, B).

### Silencing KIF3B enhances the radiosensitivity of ESCC and attenuates the radiation resistance caused by YY1 overexpression

**Fig. 4:**
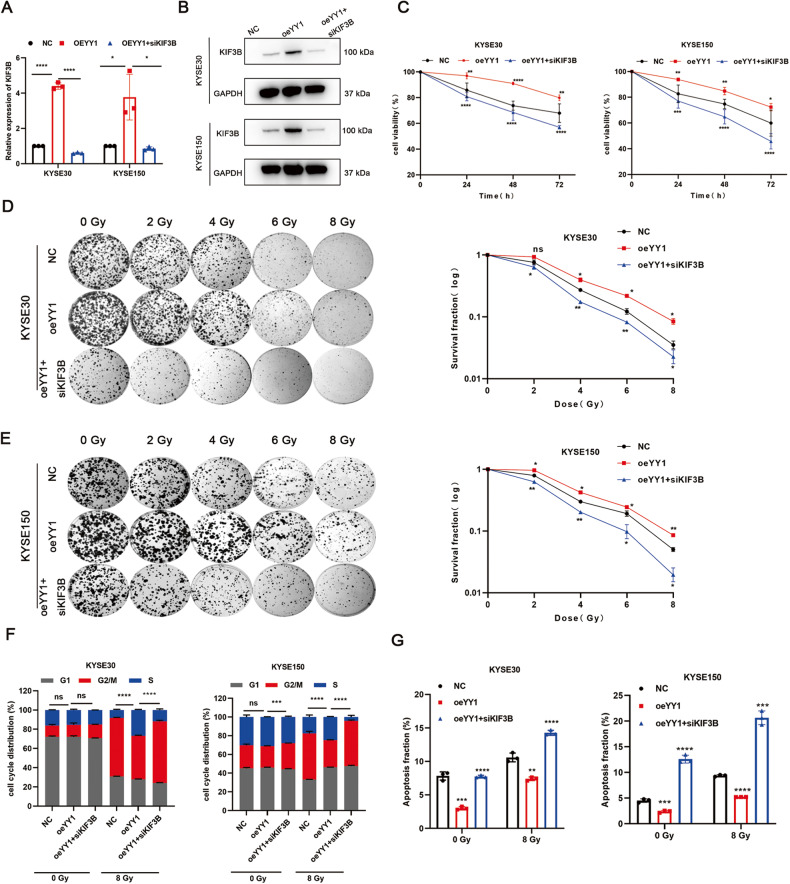
Downregulating KIF3B can alleviate radioresistance caused by YY1 overexpression. **A**, **B** Verifying efficacy via RT-qPCR and WB. **C** CCK-8 assay was used to compare cell viability. **D**, **E** Representative images of cell colonies and corresponding survival fraction. **F** Cell cycle distribution in indicated groups. **G** Apoptosis fraction in different groups. *P < 0.05, **P < 0.01, ***P < 0.001, ****P < 0.0001, ns no significance.

Afterwards, we analyzed the effect of knocking down KIF3B on the radiosensitivity of ESCC. Lentiviruses were designed to downregulate KIF3B in ESCC cells (Fig. S3C, D). CCK-8 and clonogenic assays revealed that KIF3B depletion decreased cell viability and survival fraction (Fig. S3E–H). Flow-cytometry showed knocking down KIF3B increased the number of cells in the G2/M phase and the fraction of radiation-induced apoptotic cells (Fig. S4A–F). Besides, we also found similar results in vivo, tumor volume and weight decreased after RT in shKIF3B groups obviously (Fig. S4G–I). To confirm whether YY1 modulated ESCC radiosensitivity through transcription of KIF3B, we carried out the following rescue experiments. Firstly, we transfected siKIF3B in YY1 overexpressed cells and verified the efficiency (Fig. 4A, B). CCK-8 and colony formation assays revealed that silencing KIF3B alleviated the increased proliferation rate caused by overexpressing YY1 (Fig. 4C–E). Flow-cytometry showed KIF3B knockdown can reverse reduced G2/M arrest and apoptotic fraction due to YY1 upregulation (Fig. 4F, G, Fig. S5A–D). These results suggested that YY1 regulates the radiosensitivity of ESCC cells in a KIF3B-dependent manner.

### YY1 influences the radiosensitivity of ESCC through KIF3B-mediated Hippo signaling pathway

**Fig. 5:**
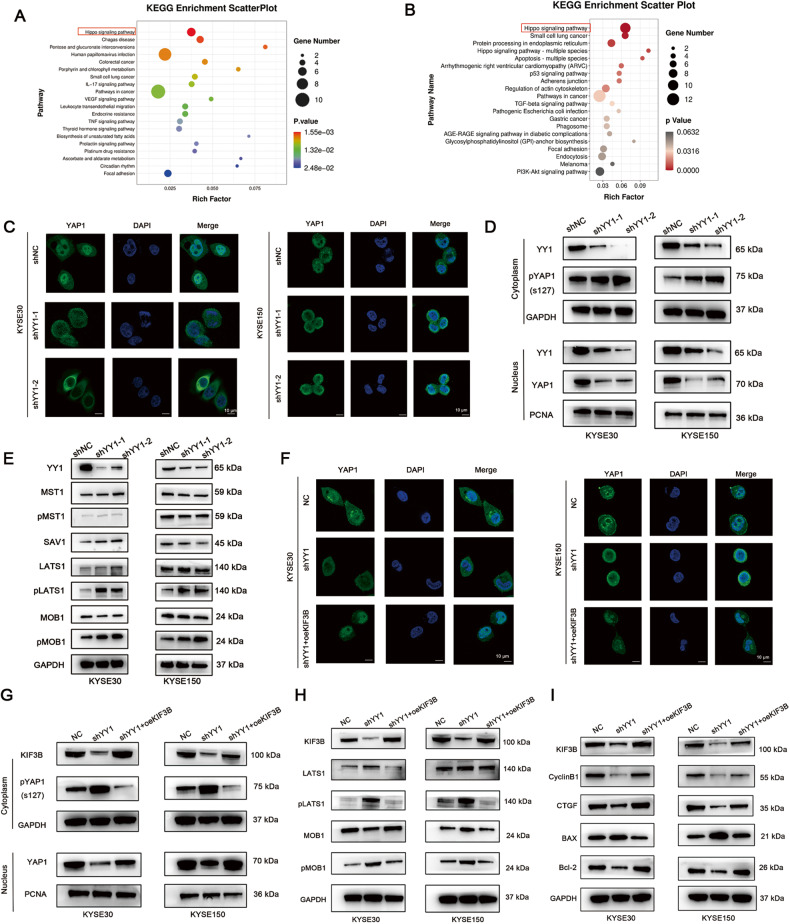
YY1 influences the radiosensitivity of ESCC through KIF3B-mediated Hippo pathway. **A**, **B** KEGG plots displayed the enrichment of signaling pathway after silencing YY1 **A** and KIF3B **B**. **C** Representative IF images showed the localization of YAP1 in shNC and shYY1 groups. Scale bar, 10 μm. **D** WB assay displayed the expression of YAP1 in the cytoplasm and the nucleus. **E** The expression of the main components of Hippo signaling were tested by WB. **F** Representative IF images showed the localization of YAP1 in different groups. Scale bar, 10 μm. **G**–**I** WB assay was performed to detect the expression of YAP1 in the cytoplasm and the nucleus, the expression of components and target genes of the Hippo pathway.

To elucidate the mechanisms underlying YY1 and KIF3B mediated downstream signaling, we analyzed the results of the Kyoto Encyclopedia of Genes and Genomes (KEGG) analysis after depleting YY1 and KIF3B. We found that the Hippo signaling pathway was activated significantly (Fig. 5A, B). Reportedly, upon activation of Hippo signaling, its main effector, YAP1 is phosphorylated on Ser127, which further inhibits its transcriptional activity in the nucleus [22].Therefore, we carried out IF and subcellular fractionation assays to analyze the level of YAP1 expression in the nucleus. As shown in Fig. 5C, silencing YY1 suppressed the level of YAP1 in the nucleus and increased the phosphorylation of YAP1 in the cytoplasm. The nuclear-cytoplasmic separation assay also displayed the same results (Fig. 5D). YAP1 is a transcriptional co-activator and correlated with tumor progression and radioresistance [18–20]. Rescue experiments showed overexpressing YAP1 can reduce radiosensitivity caused by YY1-depletion (Fig. S6A–F). Moreover, after depleting YY1, the phosphorylation levels of the core Hippo signaling pathway components (LATS1 and MOB1) increased while the expression of MST1 and SAV1 did not change obviously (Fig. 5E). Considering KIF3B is the target of YY1 and KIF3B knockdown also influences the Hippo pathway, we hypothesized that YY1 may regulate Hippo signaling through KIF3B, hence we performed rescue experiments. Our results revealed that overexpressing KIF3B in shYY1 cells increased the expression of YAP1 in nucleus and inhibited the activation of the Hippo signaling pathway (Fig. 5F–H). Moreover, compared to knocking down YY1, upregulating KIF3B could lead to opposite expression of YAP1 target genes, including CTGF, Bcl-2, CyclinB1, and BAX (Fig. 5I). These results showed that YY1 regulates the radiosensitivity of ESCC cells through KIF3B-mediated Hippo signaling pathway.

### KIF3B regulates Hippo signaling pathway by interacting with ITGB1

**Fig. 6:**
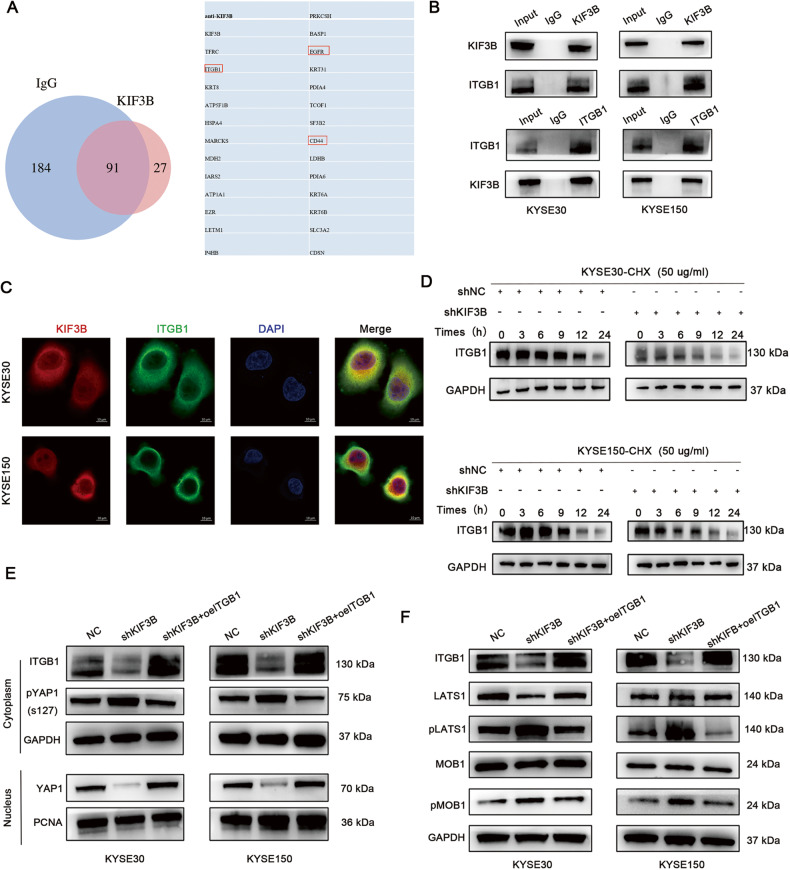
KIF3B influenced Hippo signaling pathway by interacting with ITGB1. **A** IP-MS identified the potential interacting proteins of KIF3B. **B** IP experiments validated the interaction between KIF3B and ITGB1. **C** Representative IF images showed the localization of KIF3B and ITGB1. Scale bar, 10 μm. **D** WB analysis showed that KIF3B silencing increased the degradation of ITGB1 after treatment with CHX (50 μg/ml). **E**, **F** WB assay displayed the expression of YAP1 and the main components of the Hippo pathway.

**Fig. 7:**
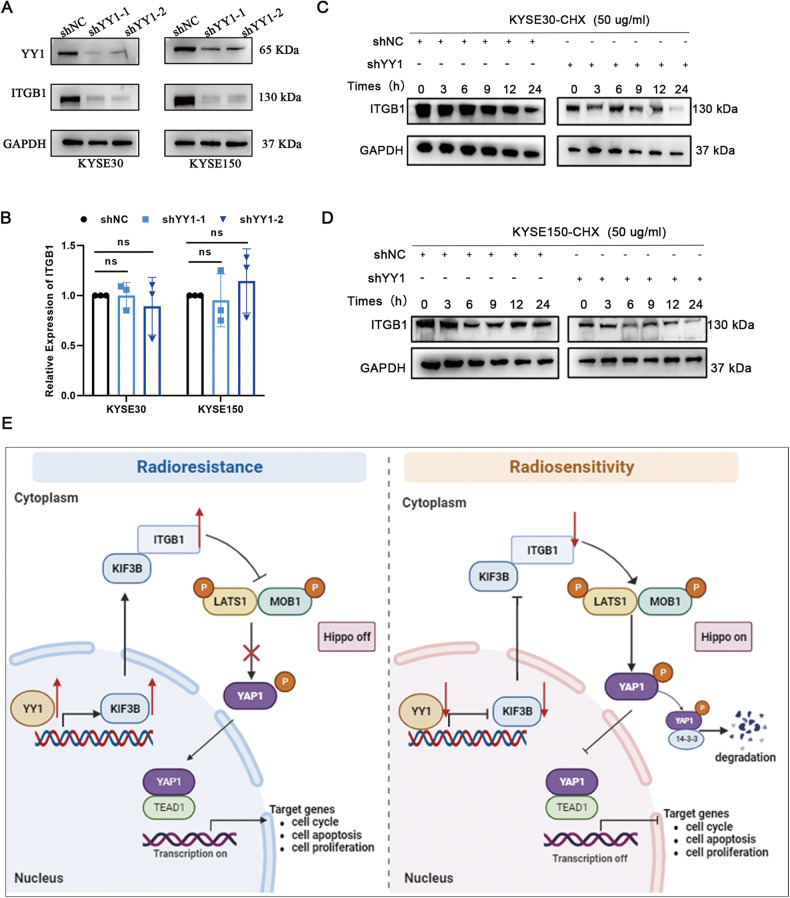
Correlation between YY1 and ITGB1 and the mechanism of regulatory network of YY1 in influencing radiosensitivity of ESCC. **A**, **B** WB and RT-qPCR assays showed the protein and mRNA level of ITGB1 in shNC and shYY1 groups. **C**, **D** WB assay showed that YY1 depletion accelerated the degradation of ITGB1 after treatment with CHX (50 μg/ml). **E** The mechanism of regulatory network of YY1 in influencing radiosensitivity of ESCC. ns: no significance.

Hippo signaling pathway can be regulated by a variety upstream factor, however, since there was no direct evidence suggesting KIF3B as the direct upstream regulator of Hippo pathway, we hypothesized that there was a mediator between KIF3B and the Hippo pathway. Therefore, we performed IP-MS analysis to identify this mediator protein (Fig. S6G). As shown in Fig. 6A, we identified a KIF3B-interacting protein network, including ITGB1, EGFR and CD44, which have been previously reported to be the upstream factor of Hippo pathway previously [23–25]. Finally, we selected ITGB1 as the candidate protein, because IP assay confirmed KIF3B could co-immunoprecipitated with ITGB1 in ESCC cells (Fig. 6B). Subcellular localization analysis revealed that these two proteins co-localized in the cytoplasm (Fig. 6C). The protein stability assay showed that silencing KIF3B greatly accelerated the degradation of ITGB1. These results indicated that the interaction between KIF3B and ITGB1 stabilized each other in the cytoplasm (Fig. 6D, Fig. S6H, K). Moreover, the expression of ITGB1 correlated with radiosensitivity in our clinical samples (Fig. S7A). Hence, we also explored the effect of silencing ITGB1 on the radiosensitivity of ESCC in vitro and in vivo. Lentiviruses were designed to knockdown ITGB1 in ESCC cells (Fig. S7B, C). CCK-8 and clonogenic assay showed that ITGB1 downregulation decreased cell viability and survival fraction (Fig. S7D–G). Animal experiments also showed tumor volume and weight decreased after X-ray irradiation in ITGB1 depletion groups (Fig. S7H–J). To further verify whether KIF3B influences Hippo pathway through ITGB1, we conducted recovery experiments and results showed that ITGB1 overexpression in shKIF3B cells could inhibit the activation of the Hippo signaling pathway (Fig. 6E, F), thus confirming that KIF3B regulated the Hippo pathway through interaction with ITGB1. Moreover, we also explored the YY1 depletion effect on ITGB1 mRNA and protein stability, we found the protein level of ITGB1 decreased in YY1-depletion groups (Fig. 7A). However, knocking down YY1 had no effect on the mRNA level of ITGB1, which demonstrated YY1 could not regulate the transcription of ITGB1 (Fig. 7B). The protein stability assay showed that silencing YY1 accelerated the degradation of ITGB1 (Fig. 7C, D, Fig. S7K).

Overall, our study revealed that silencing YY1 can enhance ESCC radiosensitivity through KIF3B-mediated Hippo signaling pathway (Fig. 7E).

## Discussion

Recently, nCRT has been recommended as the standard treatment for advanced ESCC. Comparing with surgery alone, nCRT has a better R0 resection rate, longer survival rates, and a lower recurrence rate [26, 27]. However, its therapeutic efficacy still remains limited due to the high intrinsic radioresistance in ESCC [28]. Our study revealed that increased expression of YY1 could lead to poor response rate and shorter survival in patients with ESCC receiving nCRT. Based on the results of CCK-8 and colony formation assays, we found that cell proliferation decreased significantly after RT, especially in YY1-depleted groups. Moreover, flow-cytometry showed that silencing YY1 promoted cell cycle arrest and increased apoptotic cells after RT. Consistently, YY1 knockdown also enhanced radiosensitivity of ESCC cells in our mice model. Thus, YY1 may be a potential biomarker and therapeutic target for modulating the radiosensitivity of ESCC.

Mechanistically, using multigroup sequencing we validated that YY1 could bind to the promoter region of KIF3B and regulate transcription of KIF3B. KIF3B is a molecular motor protein and consists of three domains: motor domains, PH, and FHA domains [29]. Conserved motor domains are responsible for connecting microtubules and ATP through the specified binding site, PH domains participate in transporting cargos and little is known about the FHA domains [30]. Recently, increasing attention has been paid to the relativity between KIF3B and the occurrence of tumors [31]. Our study is the first to find that radiotherapy-treated ESCC cells with low KIF3B expression, show markedly impaired cell viability and proliferation rates in vitro and in vivo. Furthermore, patients with ESCC with lower expression of KIF3B could benefit more from nCRT.

Besides, we identified that both YY1 and KIF3B modulated the Hippo signaling pathway. Functional experiments verified that YY1 regulated the Hippo pathway through regulating the expression of KIF3B. Inhibiting KIF3B transcription activated the Hippo pathway and decreased the level of YAP1 expression in the nucleus. This in turn, reduced the transcription of numerous crucial genes that are involved in DNA repair and proliferation, facilitating reduced tolerance of tumor cells to IR-induced cytotoxicity and finally making them more radiosensitive [32].

Based on the function and structure of KIF3B, we hypothesized that there were mediator proteins between KIF3B and the Hippo pathway. Hence, we performed MS analysis to better understand how KIF3B regulates the Hippo signaling pathway. We selected the candidate protein ITGB1 which can interact with KIF3B. ITGB1 is an integrin and plays an important role in tumor progression and in the constitution of the cytoskeleton [33], where KIF3B is involved in intracellular transportation, mitosis, and cell formation which is dependent on cytoskeleton structures [30], Our IP and IF assays confirmed that KIF3B interacts with ITGB1. Moreover, previous studies reported that ITGB1 could influence DNA damage repair and radiosensitivity of breast and non-small lung cancer cells via regulating Hippo-YAP1 signaling pathway [23, 34]. These findings prompted us to validate whether KIF3B could influence Hippo pathway through interaction with ITGB1. Our rescue experiments showed that overexpression of ITGB1 in KIF3B-depletion cells increased the level of YAP1 expression in the nucleus and inhibited phosphorylation of the main components of the Hippo pathway, which contributed to pathway and hampered the radiosensitivity in ESCC.

In conclusion, our study revealed that YY1 was upregulated in patients unresponsive to neoadjuvant therapy, and suppression of YY1 inhibited the transcription of KIF3B, which resulted in the activation of the Hippo signaling pathway through interaction with ITGB1. Once the pathway was activated, the expression of target genes such as CTGF, Bcl-2, and CyclinB1 could be inhibited, which in turn strengthened the radiosensitivity in ESCC. The current study provides new insights into the mechanisms underlying ESCC radioresistance and highlights the potential of YY1 as a therapeutic target for ESCC.

## Materials and methods

### Human participants

We collected 50 endoscopic specimens from patients with ESCC before undergoing nCRT at Shandong Cancer Hospital between January 2020 and May 2022. None of these patients received any antitumor therapy prior to nCRT. Evaluating the efficiency of nCRT according to the tumor regression grade (TRG) based on the College of American Pathologists system (available at http://www.cap.org). Samples used in this study were authorized by the Ethics Committee of Shandong Cancer Hospital and Institute.

### Immunohistochemistry (IHC) staining

Endoscopic specimens (4 μm) embedded with paraffin were used for IHC staining. The procedure was performed as previously described [35]. Antibodies used in IHC are shown in Supplementary Table S1. Two independent pathologists assessed the outcomes according to Histochemistry score (H-score), which was calculated by multiplying the proportion of positive cells by staining intensity. The staining intensity was graded as negative (0), weak (1), moderate (2), and strong staining (3) [36]. According to the cutoff of median expression, H-score ≥ 200 was determined as YY1-high expression and samples with H-score <200 was defined as YY1-low expression. Similarly, H-score ≥ 110 was regarded as KIF3B-high expression while H-score <110 was defined as low expression.

### Cell lines and cell culture

HEK293T and human ESCC cell lines were grown in Dulbecco’s modified Eagle’s medium (Gibco, Thermo Fisher Scientific Inc., Chino, CA, USA), containing 10% fetal bovine serum (FBS, Gibco, CA, USA) and 1% penicillin-streptomycin solution (Biosharp, Hefei, China). Cells were authenticated by STR profiling (Shanghai, China) and cultured at 37 °C in a 5% CO_2_ incubator.

### Lentivirus transduction and transfection of small interfering (si) RNAs and plasmids

Lentiviruses were designed by Genechem (Shanghai, China), while plasmids and siRNAs were obtained from GenePharma (Shanghai, China). Lentiviruses were added to ESCC cells when the cell density reached 30–40%, and puromycin (Solarbio, Beijing, China) was used to select the stably transfected cells. siRNAs were transfected to cells using the siRNA Mate solution (Genepharm, Shanghai, China), siRNA sequences are shown in Supplementary Table S2. Plasmids were transfected into cells using Lipofectamine 3000 reagent (Invitrogen, Carlsbad, CA, USA), when the cell density reached 70–80%.

### Reverse transcription‑quantitative PCR (RT‑qPCR)

Isolating RNA from cells using TRIzol reagent (Invitrogen, Carlsbad, CA, USA) following the manufacturer’s instructions. Each RNA sample was reverse transcribed into cDNA using the PrimeScript™ RT Master Mix (Takara, Japan) and RT-qPCR was performed using TB Green Premix Ex Taq II (Takara, Japan). The primer sequences used in this study are listed in Supplementary Table S2.

### Protein extraction and Western blotting (WB)

Total protein was extracted using RIPA buffer (Beyotime, Shanghai, China) while nuclear protein was extracted using the Nuclear Protein Extraction Kit (Solarbio, Beijing, China) following the manufacturer’s instructions. Then protein concentrations were determined and protein samples were subjected to SDS-PAGE. After transferring to PVDF membranes and blocking with 5 % milk, the membranes were incubated overnight at 4 °C using the antibodies shown in Supplementary Table S1. Finally, the bands were visualized the bands using ECL substrate (Millipore, Darmstadt, Germany).

### X-ray irradiation

Cells and mice were irradiated with X-RAD 225 irradiator (Rad Source, Suwanee, GA, USA). The voltage of the irradiator was 225 KV and the dose rate was 200 cGy/min.

### Cell Counting Kit (CCK)-8 and colony formation assays

CCK-8 assay was performed to detect cell viability. ESCC cells (2000/well) were seeded into 96-well plate, after 16–24 h incubation, RT group was irradiated with 8 Gy X-ray. 10 μL of CCK-8 solutions (Bioss, Beijing, China) were added to the 96-well plate and incubated for 2 h without light. Absorbance in each well was measured at 450 nm. For colony formation assays, cells were inoculated into 6-well plates at a density of 1000 cells/well and irradiated to 0, 2, 4, 6, 8 Gy respectively after attachment. Cells were collected for another 7–14 days post radiation. Colonies were stained with crystal violet (Bioss, Beijing, China), numbers of cell colonies were counted and finally photographed.

### Flow cytometry analysis

To analyze the distribution in the cell cycle, ESCC cells were harvested at 24 h post 8 Gy and then fixed with 70% ethanol for 72 h. The cells were stained with cell cycle detection kit containing propidium iodide (PI, Beyotime, Shanghai, China) for 30 min without light, and then analyzed by flow cytometry (BD Biosciences, Franklin Lakes, NJ, USA). Cells were gathered at 48 h after 8 Gy irradiation and stained with the FITC/Annexin-V (BD Biosciences, Franklin Lakes, NJ, USA) for analysis of apoptosis.

### ESCC xenografts

Five-week-old nude mice (Vital River Laboratory, Beijing, China) were used to construct the tumor model. Briefly, we injected 3 × 10^6^ ESCC cells into the right flanks of nude mice. Experiments involving mice were approved by the Shandong Cancer Hospital. Mice were divided randomly into RT group and non-RT (NRT) group after injecting 7–10 days (*n* = 4 per group). RT group was conducted at 8 Gy dose for 2 times using an X-ray irradiator. Tumor volumes were calculated according to the following formula: Volume = 0.5 × length × width^2^.

### RNA-sequencing and ChIP-sequencing

Total RNA was extracted with TRIzol reagent. Library construction and sequencing were performed on an Illumina Novaseq 6000 (LC-Bio Technology Co., Ltd, Hangzhou, China) following the manufacturer’s protocol. For ChIP-sequencing, cells were harvested, crosslinked, and sonicated, following which immunoprecipitation was carried out using anti-YY1.

### Dual‑reporter assays

About 2 × 10^4^ HEK293T cells /well were inoculated into 96-well plates. Plasmids carrying YY1, pGL3-wild type KIF3B, pGL3-mutant KIF3B, pGL3-NC and Renilla luciferase plasmids (GenePharma, Shanghai, China) were co-transfected into cells using Lipofectamine 3000 Reagent. Luciferase activity was estimated at 48 h after transfection using the Dual-Glo Luciferase Assay System (Promega, Madison, USA) on a SpectraMax (Thermo Fisher, Chino, CA, USA).

### Immunofluorescence (IF)

Cells were first fixed in paraformaldehyde, followed by permeabilization and blocking, after which cells were incubated with primary antibodies overnight (Supplementary Table S1). Cells were then incubated with secondary antibody for 1 h, and subsequently stained with DAPI (Beyotime, Shanghai, China) for 15 min. Images were visualized and recorded using a confocal laser scanning microscope (Zeiss, Jena, Germany).

### Immunoprecipitation (IP) and mass spectrometry (MS)

Lysed cells were subjected to immunoprecipitation with IgG or the anti-KIF3B antibody using the IP Kit (Dai-an, Wuhan, China) according to the manufacturer’s protocol. Then strips were subjected to MS analysis to recognize KIF3B-interacting proteins. Q-Exactive (Thermo Fisher, Chino, CA, USA) was used as a mass spectrometer to identify proteins. Briefly, proteins were quantified firstly, the three-dimensional structure of the proteins was opened through reductive alkylation, the peptide segments were exacted after enzymatic hydrolysis. Then identifying the proteins through Proteome Discoverer software (Thermo Fisher, Chino, CA, USA). Parameters used for analysis and protein identification shown in Supplementary Table S3.

### Protein stability assay

For protein stability assays, 50 μg/mL cycloheximide (CHX, MCE, NJ, USA) was added to ESCC cells, and cells were harvested at the specified time points (0, 3, 6, 9, 12, 24 h) after addition of CHX. Then lysing cells and preparing protein samples for WB assay.

### Statistical analysis

Data were analyzed using IBM SPSS version 26.0 (SPSS, Inc, Chicago, IL, USA) and GraphPad Prism version 8.0 (GraphPad Software, Inc., CA, USA), and presented as the mean ± standard deviation (SD). Discrepancy between the two groups were quantified using Student’s t-test. Survival curves were assessed with Kaplan–Meier and the log-rank test. *P* < 0.05 was considered to be statistically significant.

### Supplementary information

Supplementary Figure Legends

Figure S1

Figure S2

Figure S3

Figure S4

Figure S5

Figure S6

Figure S7

Supplementary Tables

Original Data File

aj-checklist

## Data Availability

The data supported the findings in this study are available from the corresponding author on reasonable request.
