# Supplementary material for: YY1 modulates the radiosensitivity of esophageal squamous cell carcinoma through KIF3B-mediated Hippo signaling pathway
Source: Cell Death Dis. 2023 Dec 8;14(12):806. doi: 10.1038/s41419-023-06321-x (PMC10709558; doi:10.1038/s41419-023-06321-x)
Supplement: Supplementary file 1 — Supplementary Figure Legends [file 41419_2023_6321_MOESM1_ESM.docx]

**Supplementary Figure Legends**

**Fig. S1** **YY1 regulated the radiosensitivity of ESCC. A** Representative Images of YY1 IHC staining. Scale bar, 100 μm and 50 μm. **B** Validation YY1 antibody for IHC using WB in shYY1-ESCC cells. **C, D** CCK-8 assay showed the cell viability after RT in different groups. **E-H** Images revealed the number of cell colonies and corresponding survival fraction. **I-K** Flow cytometry assay was performed to analyze cell cycle distribution. **P*< 0.05, ***P* < 0.01, ****P* < 0.001, *****P* < 0.0001.

**Figure S2** **YY1 regulated the radiosensitivity of ESCC**. **A-D** Representative images of apoptosis in different groups. **E, F** Pictures showed the subcutaneous xenografts. **G** Volcano plots exhibited the top 20 differential genes. **P*< 0.05, ****P* < 0.001, ns: no significance.

**Fig. S3** **Knockingdown** **KIF3B upregulated the radiosensitivity of ESCC. A** Images of IHC staining. Scale bar, 200 μm and 50 μm. **B** Validation KIF3B antibody for IHC using WB in ESCC cells. **C, D** Verifying efficiency via RT-qPCR and WB. **E, F** CCK-8 assay showed the cell viability after RT in different groups. **G, H** Images revealed the number of cell colonies and corresponding survival fraction. **P*< 0.05, ***P* < 0.01, ****P* < 0.001, *****P* < 0.0001.

**Figure S4** **KIF3B regulated the radiosensitivity of ESCC.** **A-C** Flow cytometry assay was performed to analyze cell cycle distribution. **D-F** Flow-cytometry were performed to evaluate proportion of apoptosis cells. **G** Pictures showed the subcutaneous xenografts. **H, I** Tumor volume and weight of xenografts in different groups. **P*< 0.05, ***P* < 0.01, ****P* < 0.001, *****P* < 0.0001.

**Figure S5** **Representative images of cell cycle and apoptosis.** **A, B** Representative images of cell cycle distribution in indicated groups. **C, D** Representative images of apoptosis in different groups.

**Fig. S6 Upregulating YAP1 can increase radioresistance in shYY1 groups. A, B** Verifying efficacy via RT-qPCR and WB. **C, D** CCK-8 assay was used to compare cell viability. **E, F** Representative images of cell colonies and corresponding survival fraction. **G** Flow of MS analysis. **H, K** Time curves of ITGB1 degradation in ESCC cells. **P*< 0.05, ***P* < 0.01, ****P* < 0.001, *****P* < 0.0001, ns: no significance.

**Fig. S7 ITGB1 depletion sensitizes ESCC cells to RT in vitro and in vivo. A** Correlation between ITGB1 expression and TRG. **B, C** Verifying ITGB1 silencing efficiency via RT-qPCR and WB. **D, E** CCK-8 assay was performed to compare cell viability. **F, G** Colony formation assay was performed to measure cell colony formation ability after RT. **H** Picture showed the subcutaneous xenografts. **I, J** Tumor volume and weight of xenografts in different groups. **K** Time curves of ITGB1 degradation in ESCC cells. **P*< 0.05, ***P* < 0.01, ****P* < 0.001, *****P* < 0.0001.
