# Supplementary figures and images for: YY1 modulates the radiosensitivity of esophageal squamous cell carcinoma through KIF3B-mediated Hippo signaling pathway
Source: Cell Death Dis. 2023 Dec 8;14(12):806. doi: 10.1038/s41419-023-06321-x (PMC10709558; doi:10.1038/s41419-023-06321-x)

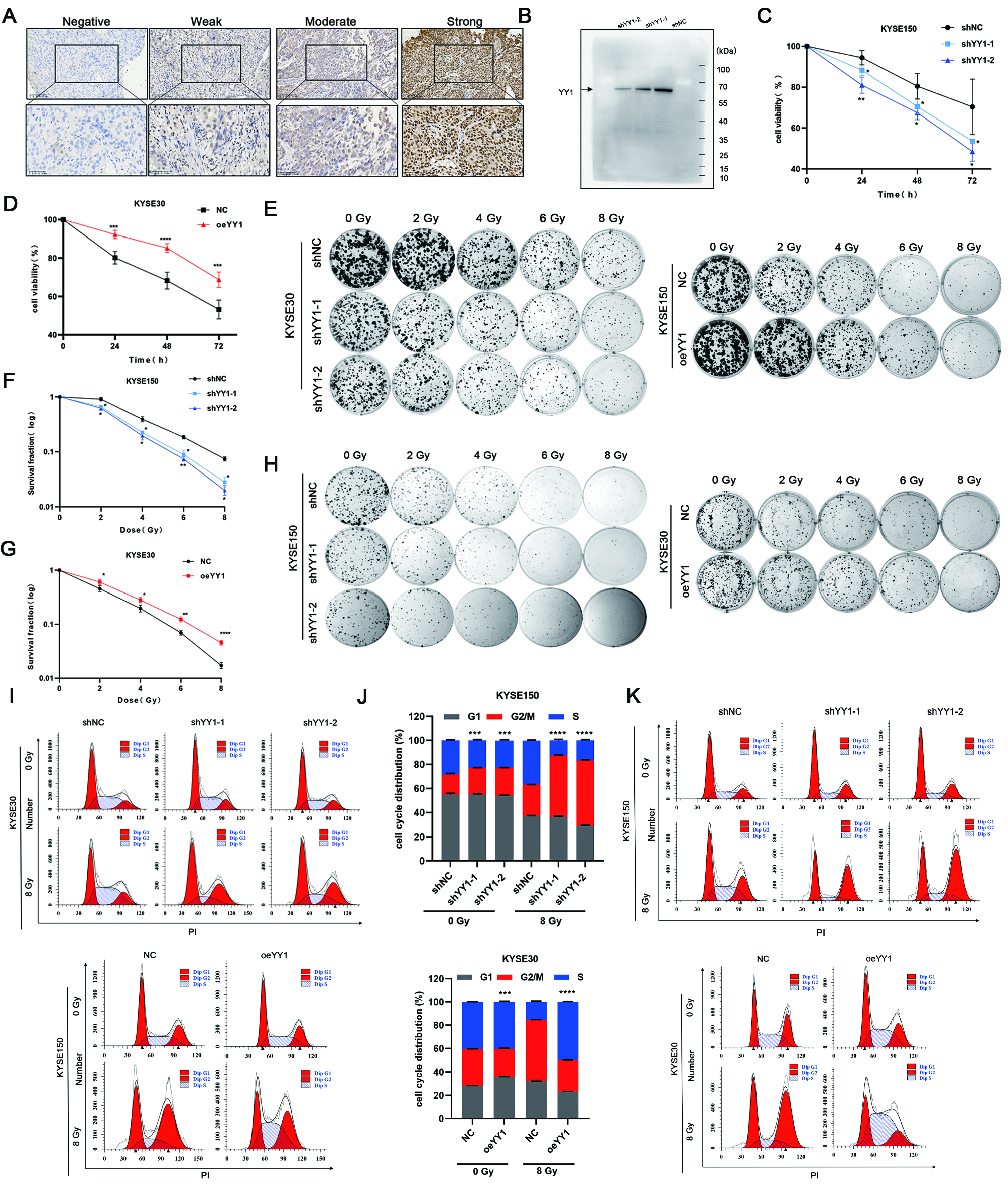

Supplement: Supplementary file 2 — Figure S1 [file 41419_2023_6321_MOESM2_ESM.tif]

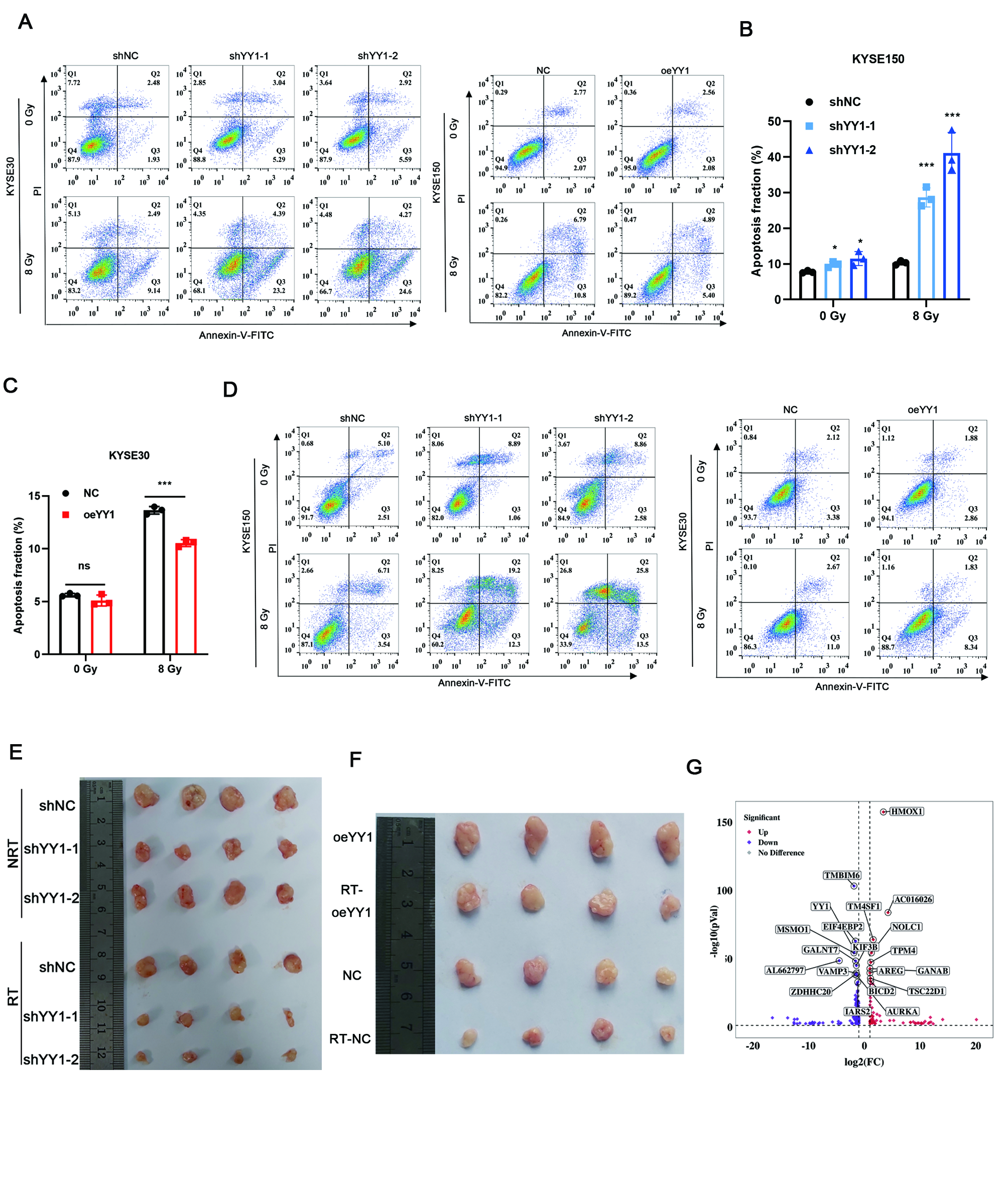

Supplement: Supplementary file 3 — Figure S2 [file 41419_2023_6321_MOESM3_ESM.tif]

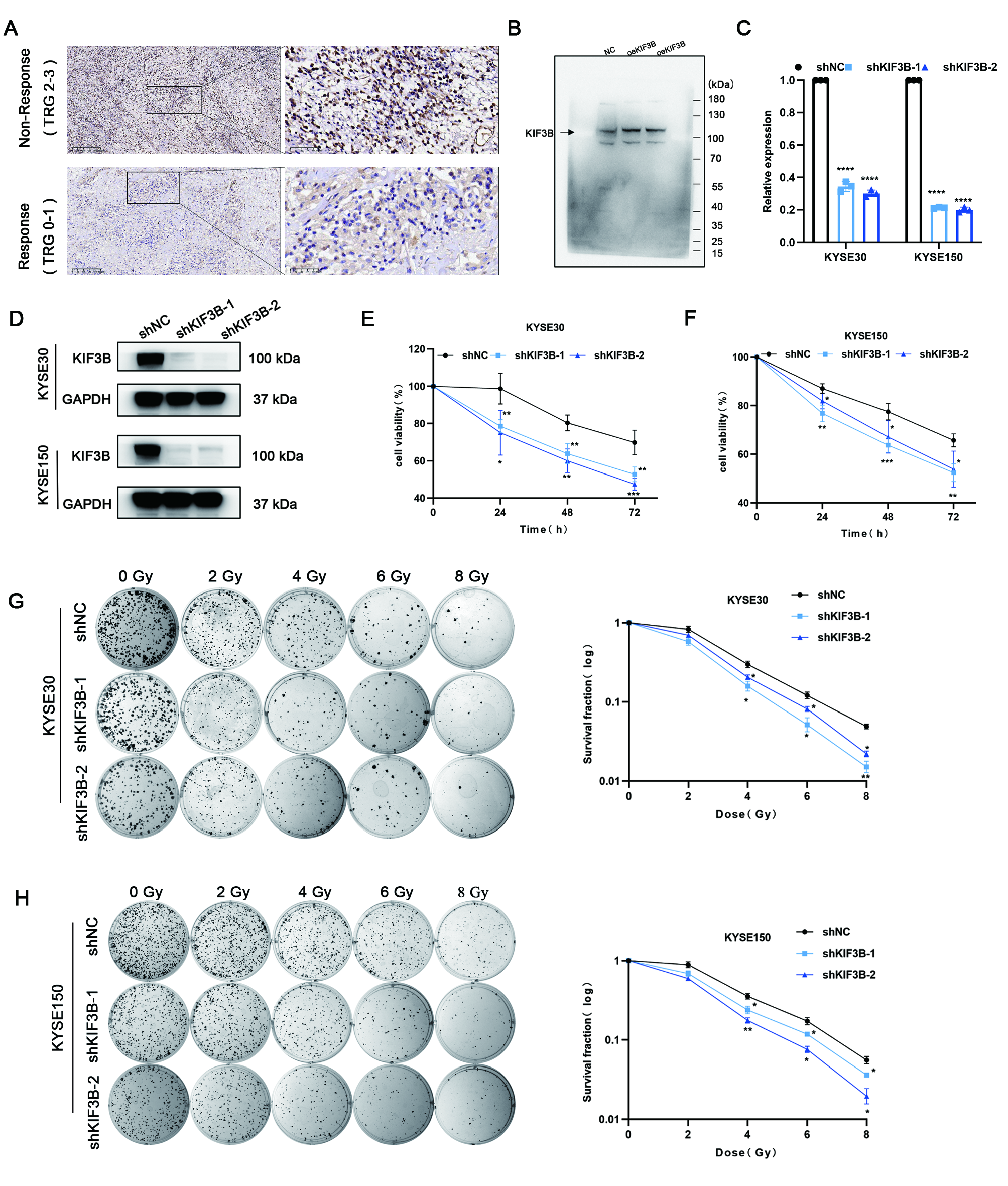

Supplement: Supplementary file 4 — Figure S3 [file 41419_2023_6321_MOESM4_ESM.tif]

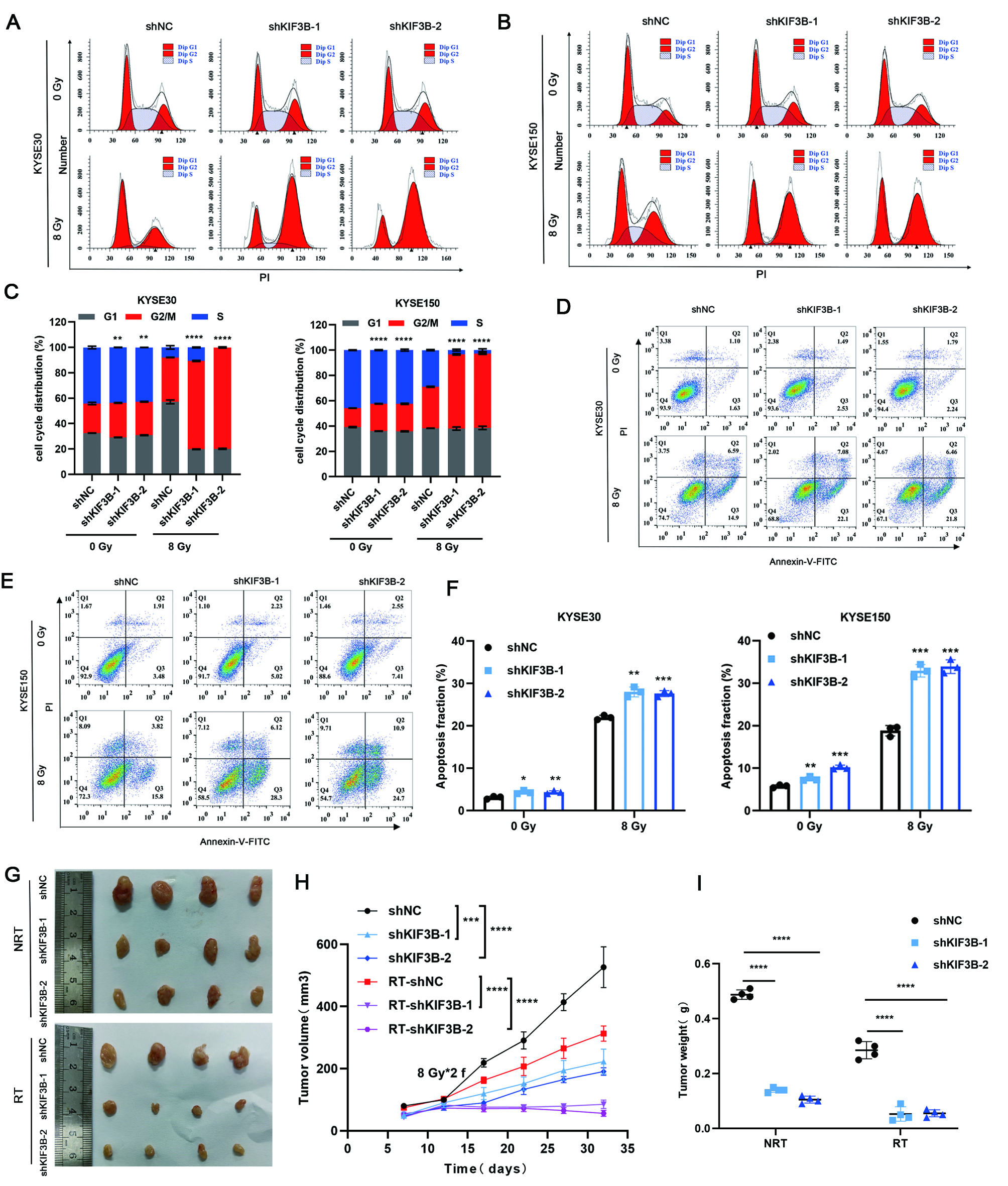

Supplement: Supplementary file 5 — Figure S4 [file 41419_2023_6321_MOESM5_ESM.tif]

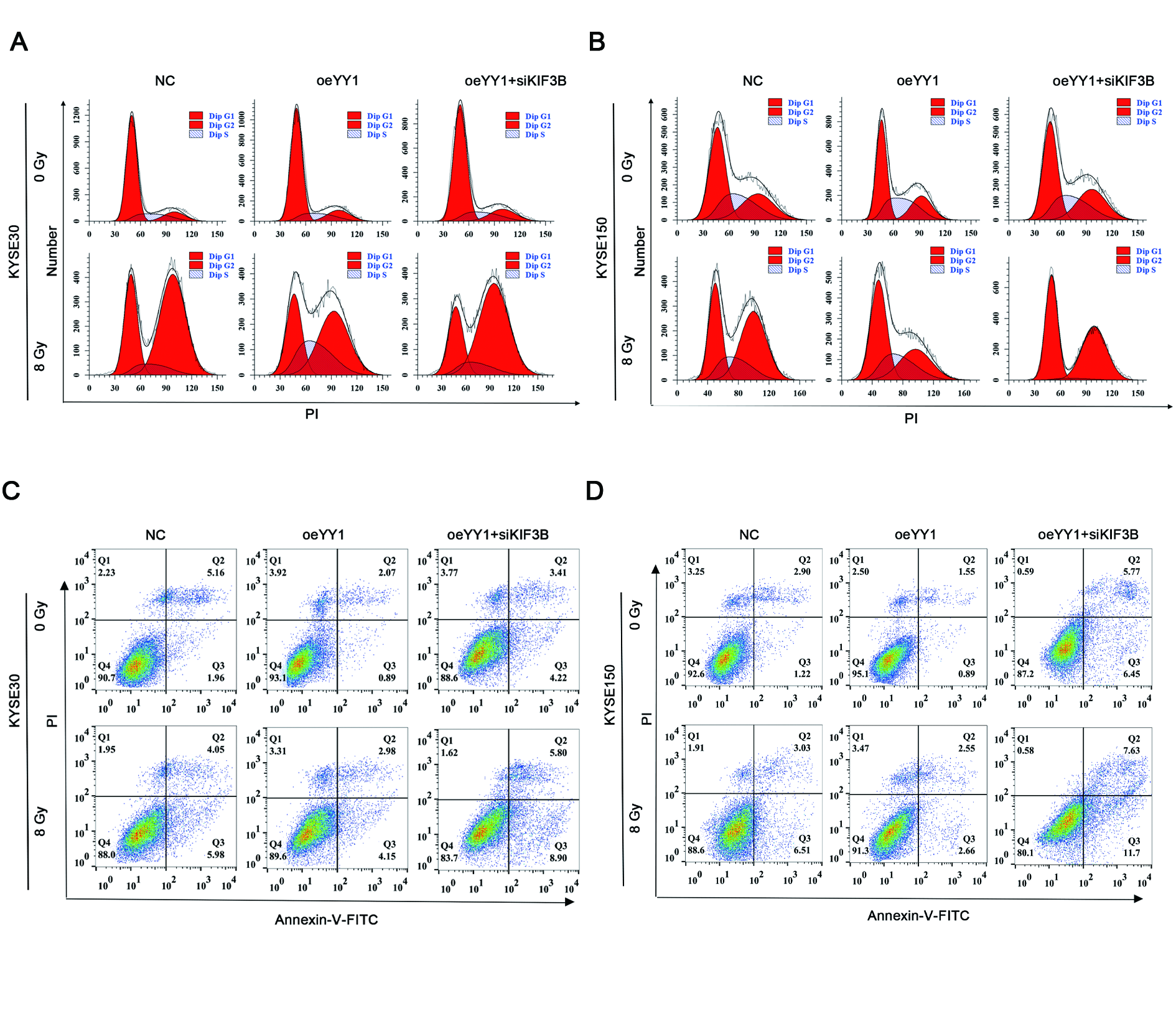

Supplement: Supplementary file 6 — Figure S5 [file 41419_2023_6321_MOESM6_ESM.tif]

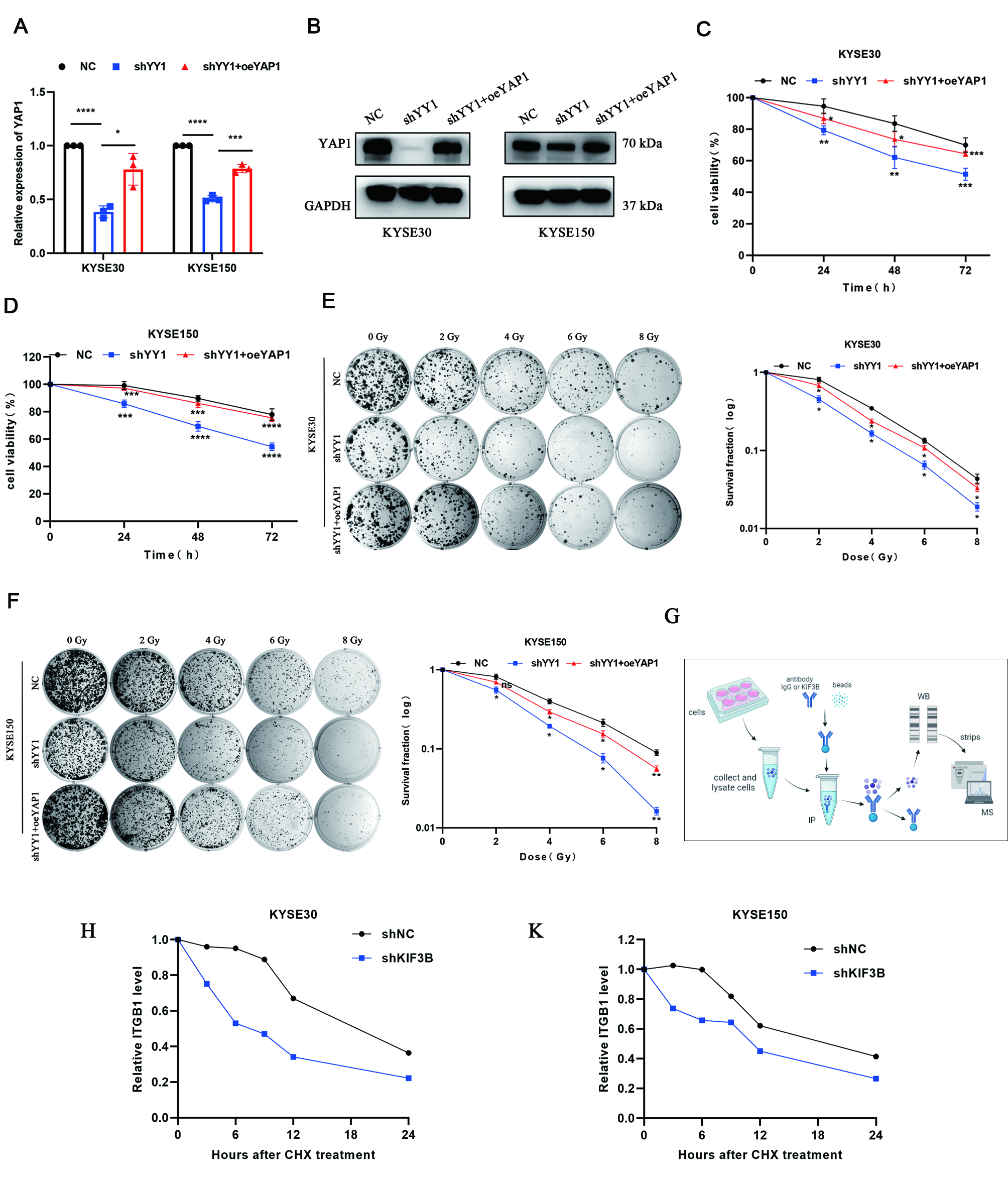

Supplement: Supplementary file 7 — Figure S6 [file 41419_2023_6321_MOESM7_ESM.tif]

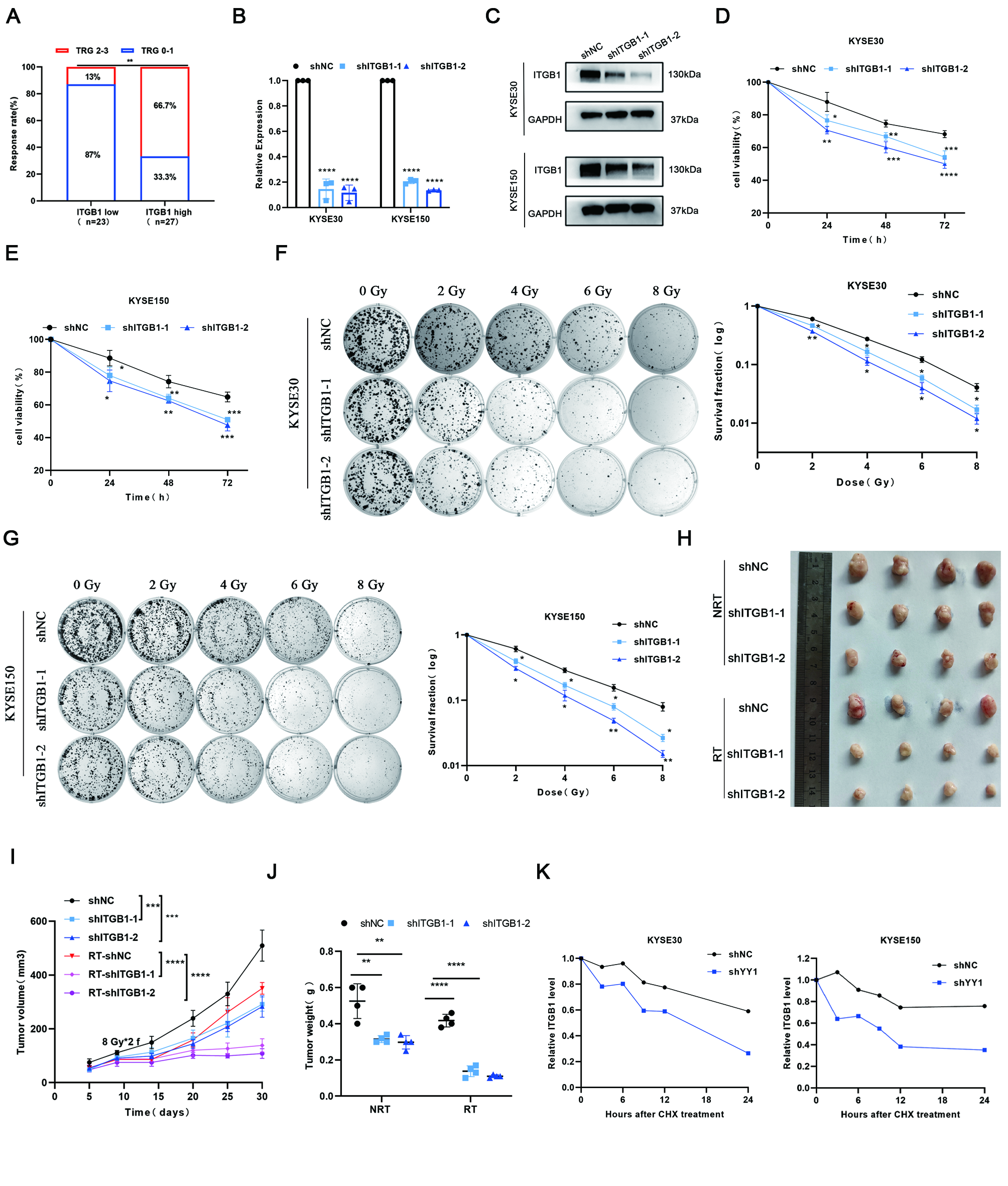

Supplement: Supplementary file 8 — Figure S7 [file 41419_2023_6321_MOESM8_ESM.tif]
