# Supplementary material for: YY1 modulates the radiosensitivity of esophageal squamous cell carcinoma through KIF3B-mediated Hippo signaling pathway
Source: Cell Death Dis. 2023 Dec 8;14(12):806. doi: 10.1038/s41419-023-06321-x (PMC10709558; doi:10.1038/s41419-023-06321-x)
Supplement: Supplementary file 9 — Supplementary Tables [file 41419_2023_6321_MOESM9_ESM.docx]

**Supplementary Tables**

**Table S1** Antibodies used in this study

| Antibodies | Dilution | Supplier | Catalogue number |
| --- | --- | --- | --- |
| YY1 | WB 1:1000 | CST | #63227 |
|  | IHC 1:400  WB 1:1000 | Proteintech  Proteintech | 22156-1-AP  22156-1-AP |
| GALNT7 | WB 1:1000 | Proteintech | 13962-1-AP |
| KIF3B | WB 1:1000 | Abclonal | A15754 |
|  | IHC 1:250 | Abclonal | A15754 |
|  | IF 1:200 | Santa Cruz | sc-514165 |
|  | IP 1-2 μg per 100-500 μg of total protein | Santa Cruz | sc-514165 |
| YAP1 | WB 1:1000 | Proteintech | 13584-1-AP |
|  | IF 1:250 |  |  |
| pYAP1(S127) | WB 1:10000 | Abcam | ab76252 |
| PCNA | WB 1:2000 | Proteintech | 10205-2-AP |
| CTGF | WB 1:1000 | Proteintech | 25474-1-AP |
| BAX | WB 1:2000 | Proteintech | 50599-2-Ig |
| Bcl-2 | WB 1:2000 | Proteintech | 12789-1-AP |
| CyclinB1 | WB 1:2000 | Proteintech | 28603-1-AP |
| GAPDH | WB 1:1000 | CST | #5174 |
| Anti-rabbit IgG, HRP-linked Antibody | WB 1:1000 | CST | #7074 |
| Goat Anti-Rabbit IgG H&L(FITC) | IF 1:1000 | Abcam | Ab6717 |
| CoraLite594-conjugated Goat Anti-Mouse IgG(H+L) | IF 1:100 | Proteintech | SA00013-3 |
| Mouse IgG | IP 1:100 | Proteintech | B900620 |
| MST1 | WB 1:1000 | CST | #3682T |
| pMST1 | WB 1:1000 | CST | #49332S |
| SAV1 | WB 1:1000 | CST | #13301T |
| LATS1 | WB 1:1000 | CST | #3477T |
| pLATS1 | WB 1:1000 | CST | #8654S |
| MOB1 | WB 1:1000 | CST | #13730S |
| pMOB1 | WB 1:1000 | CST | #8699S |
| ITGB1 | WB 1:1000  IF 1:200 | Proteintech | 12594-1-AP |

**Table S2** Sequences of the primer for RT-qPCR and siRNAs

| Assay | Sequences (5' to 3') |
| --- | --- |
| **siRNAs** |  |
| siNC | SS UUCUCCGAACGUGUCACGUTT |
|  | AS ACGUGACACGUUCGGAGAATT |
| siKIF3B | SS CCCUGCAUCUCAGCUUUAUTT |
|  | AS AUAAAGCUGAGAUGCAGGGTT |
| **shRNA** |  |
| shNC | TTCTCCGAACGTGTCACGT |
| shYY1-1 | CGGCTTCGAGGATCAGATTCT |
| shYY1-2 | CCTCCTGATTATTCAGAATAT |
| shKIF3B-1 | GGAGCAGAAACGACAGGAAAT |
| shKIF3B-2 | GGAGTCATTCCTAACTCATTT |
| shITGB1-1 | GGUAGAAAGUCGGGACAAATT |
| shITGB1-2 | CCCUCCAGAUGACAUAGAATT |
| **qRT-PCR** |  |
| YY1 | F GGATAACTCGGCCATGAGAA-3 |
|  | R ATAGGGCCTGTCTCCGGTAT |
| GAPDH | F TTCGACAGTCAGCCGCATCTTCTT |
|  | R CAGGCGCCCAATACGACCAAATC |
| HMOX1 | F GGAACTTTCAGAAGGGCCAGGT |
|  | R TGCAGCTCTTCTGGGAAGTAGACA |
| TMBIM6 | F GCGCCGTAGCTACCTCTTTCT |
|  | R GACGAAGCCACACATGACCAC |
| AC016026 | F AGCGCTGATTCTCCACCTGTT |
|  | R GGGACGACTGGGGTTTGAAGA |
| EIF4EBP2 | F TCGCAATTCTCCCATGGCTCA |
|  | R CCCCAACTGCATGTTTCCTGT |
| MSMO1 | F TGGGCATGGGTGACCATTCG |
|  | R GCCGAGAACCAGCATAGAAAGG |
| AL662797 | F TCTCTGCCTCCTTCCGTACCA |
|  | R TTGAGCTTTTCTCCTGACTGCAT |
| GALNT7 | F ACCAGTGTTTGACAAAGGGAGC |
|  | R GCAGGACCTCTGAGCGATCTA |
| ZDHHC20 | F GAAGTGGAGGTCCTCAAAGAGCA |
|  | R GCCAGTGTCTTCAGCAGATTGTT |
| IARS2 | F CAGGTCCTGACCAAAGAGCAGA |
|  | R CTCTTCCTTGCTGCCACACTTG |
| KIF3B | F GACTCTGACCACTCTGCGATATG |
|  | R TTGAGCCGAGCAATCTCTTCC |
| TM4SF1 | F ATGCCTCCGAAAACCACCTCA |
|  | R AGCCACAGCAGTCATCCTGTT |
| TPM4 | F CGTGCGGAGGTGTCTGAACTAA |
|  | R TTTCAGATGCAGCCTCCAGAGA |
| GANAB | F CTTAGCCCTCAGGGTACAGCTC |
|  | R GAATGAGAATCGACGCAGCAGG |
| AREG | F TGGTGCTGTCGCTCTTGATACT |
|  | R GTTCACGCTTCCCAGAGTAGGT |
| VAMP3 | F AGCTCTCTGAGTTAGACGACCG |
|  | R GCCCACATCTTGCAATTCTTCCA |
| AURKA | F CCATCTTCCAGGAGGACCACTC |
|  | R GGCTCCAGAGATCCACCTTCTC |
| BICD2 | F CCACCAGGTGTGACGAGTACAT |
|  | R CGTCTTCTTCTCGTCCTCAGCA |
| NOLC1 | F ATCCCCATTCCGAAGGGTCAG |
|  | R CACCTCGCTTGGCATCAAAGG |
| TSC22D1 | F GAAGTGGAGGTCCTCAAAGAGCA |
| ITGB1 | R GCCAGTGTCTTCAGCAGATTGTT  F CTCCAGAAGGTGGCTTTGATGC  R GTGAAACCCAGCATCCGTGGAA |

SS Sense strand; As Antisense strand; F Forward; R Reverse.

**Table S3** Parameters used for analysis and protein identification

| **Parameters used for analysis** |  | |
| --- | --- | --- |
| Mass spectrometer | | Q-Exactive |
| Capillary pressure | | 1.8 KV |
| Time Method duration | | 60 min |
| AGC target | | 3e6 |
| dd-MS²Resolution | | 17500 |
| Loop count  Ion source  Temperature  Full MS Resolution  Full Mass Scan range  dd-MS²Scan range  Charge exclusion | | 20  ESI  320 ℃  70000  350 to 1800 m/z  350 to 1800 m/z  1, 6 - 8, >8 |

**Parameters used for analysis and protein identification**

| Fixed modification | Carbamidomethyl |
| --- | --- |
| Variable modification | Oxidation |
| Peptide accuracy | 10ppm |
| MS/MS accuracy | 0.02 Da |
| Max missed cleavages | 2 |
| Enzyme | Trypsin |
