# Supplementary material for: YY1 modulates the radiosensitivity of esophageal squamous cell carcinoma through KIF3B-mediated Hippo signaling pathway
Source: Cell Death Dis. 2023 Dec 8;14(12):806. doi: 10.1038/s41419-023-06321-x (PMC10709558; doi:10.1038/s41419-023-06321-x)
Supplement: Supplementary file 10 — Original Data File [file 41419_2023_6321_MOESM10_ESM.docx]

**Fig 1F**

**
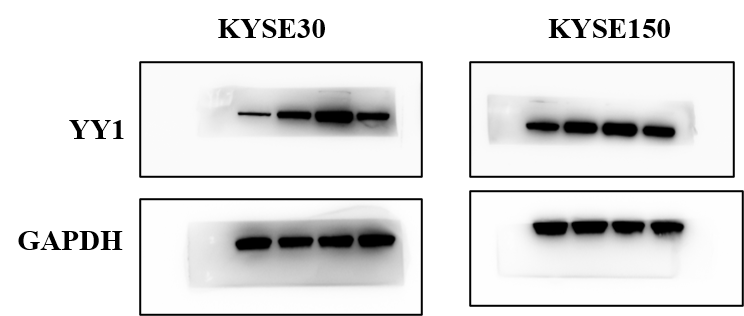
**

**Fig 2B**


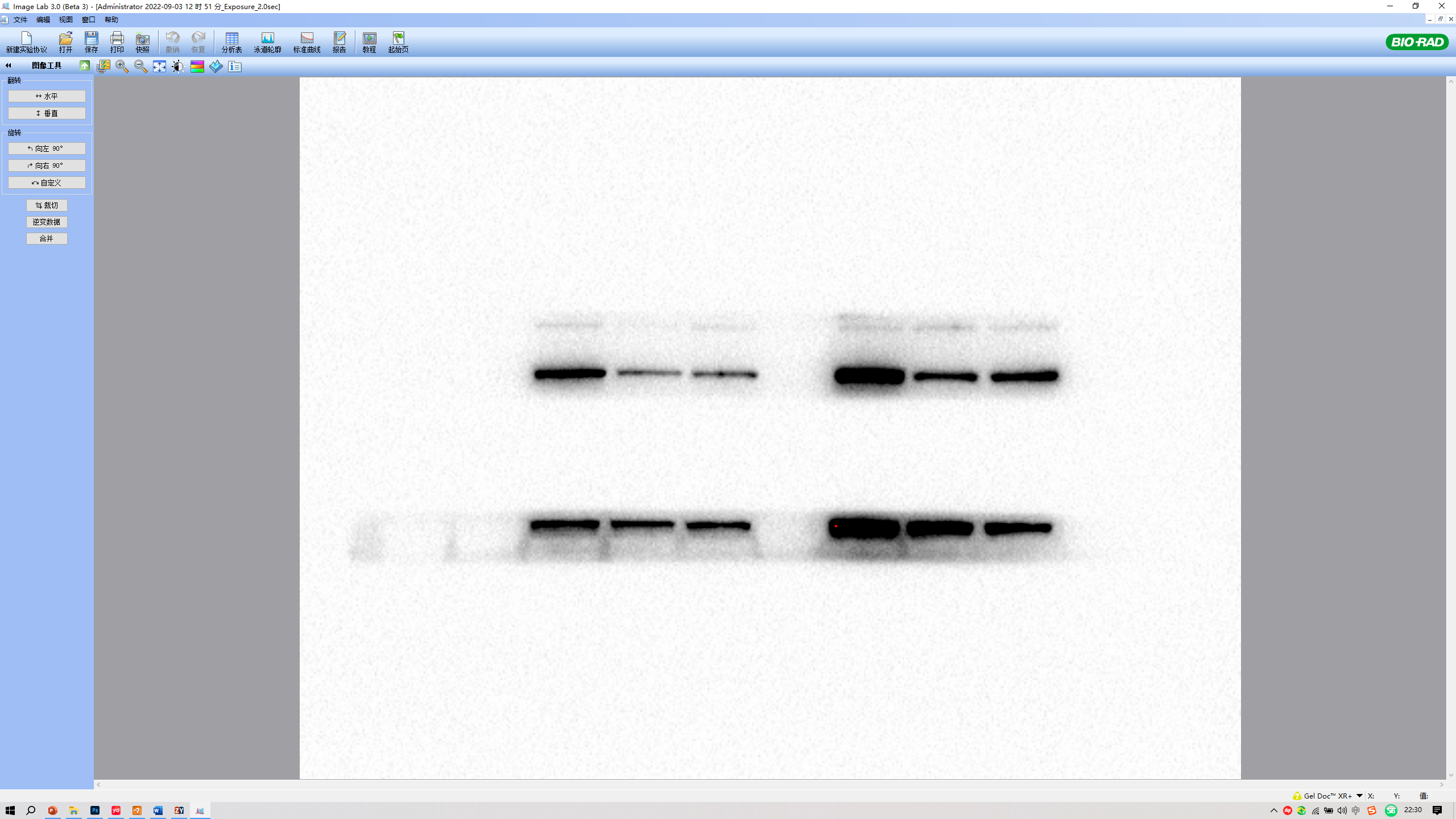

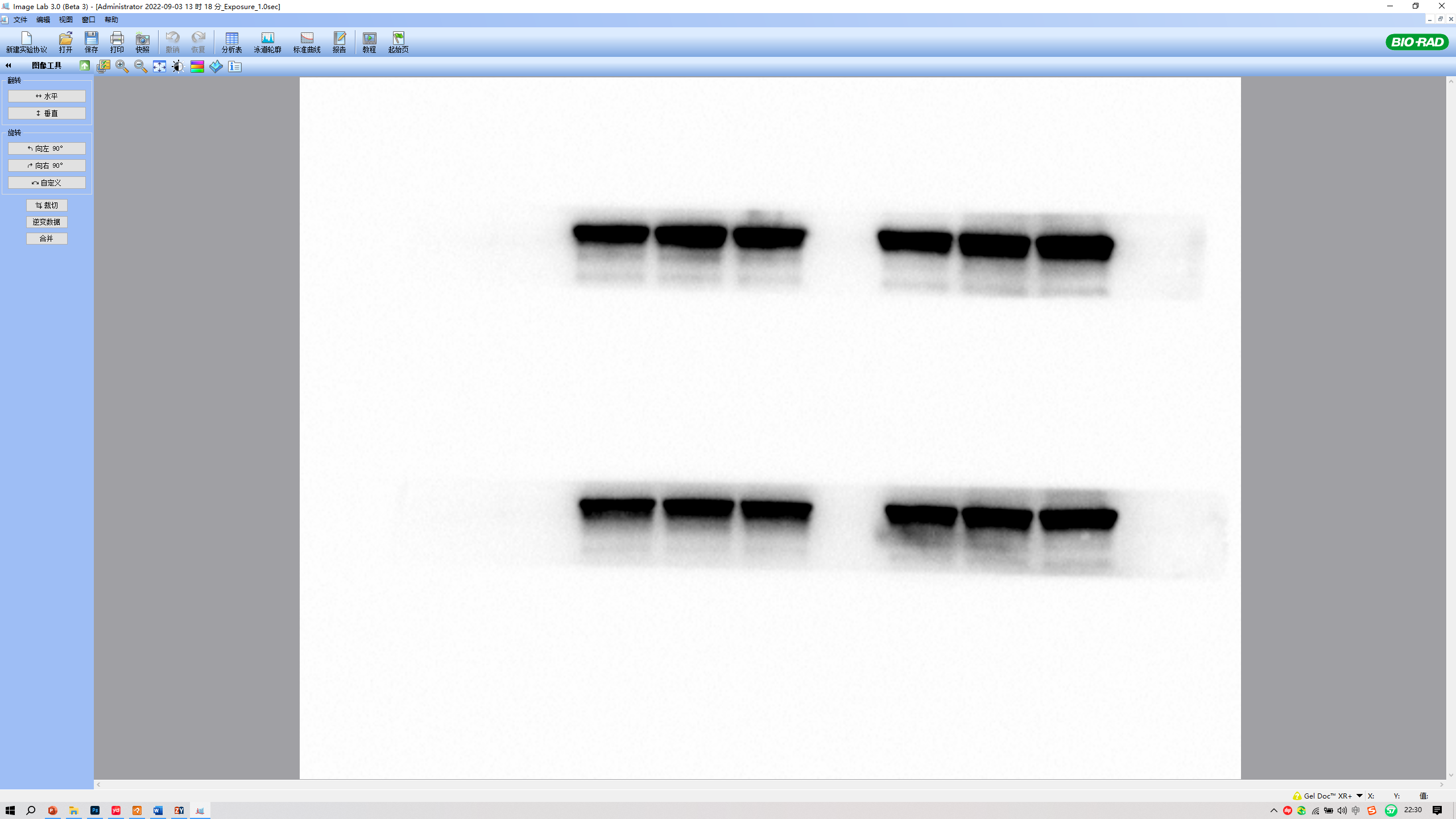

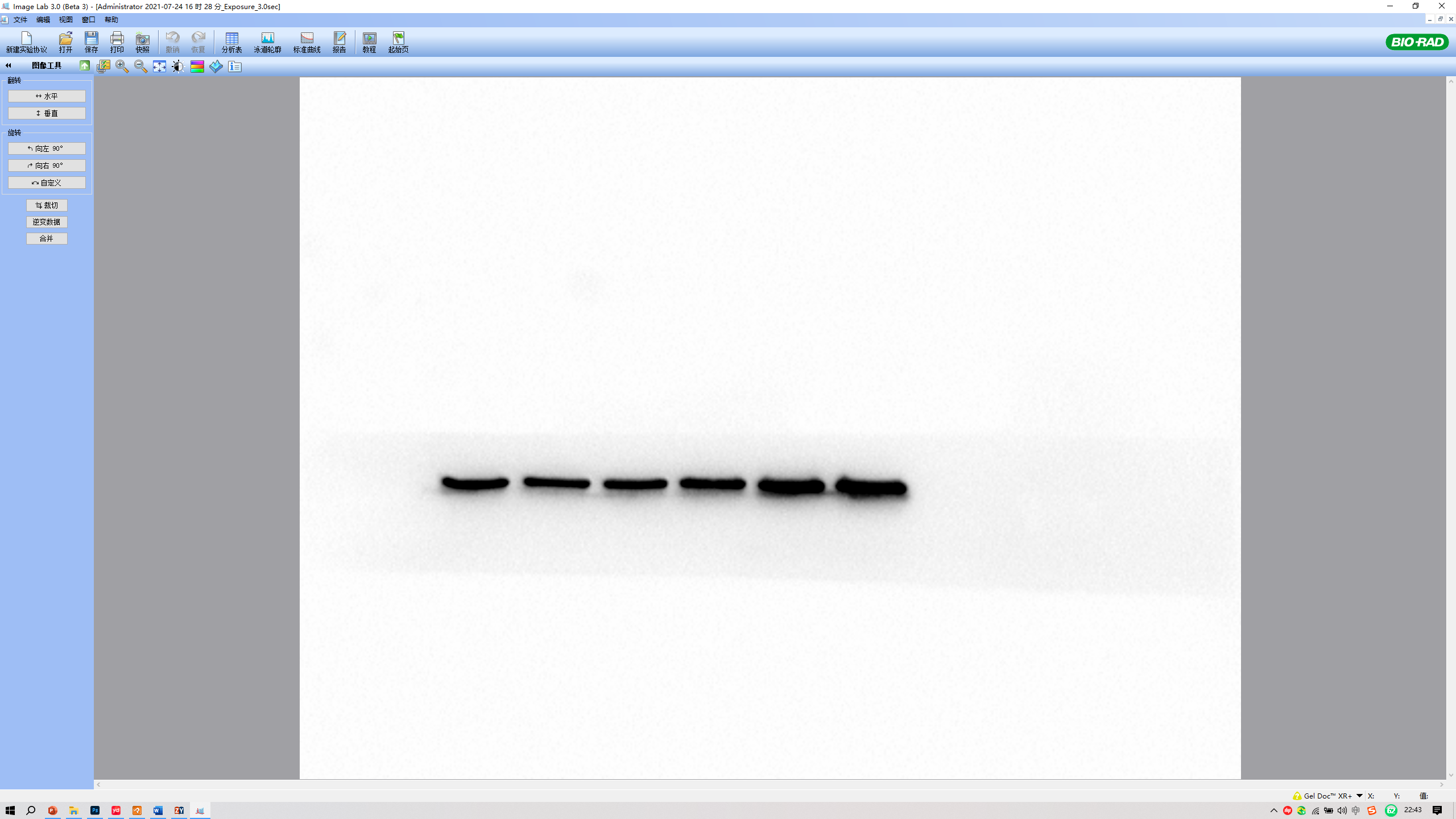

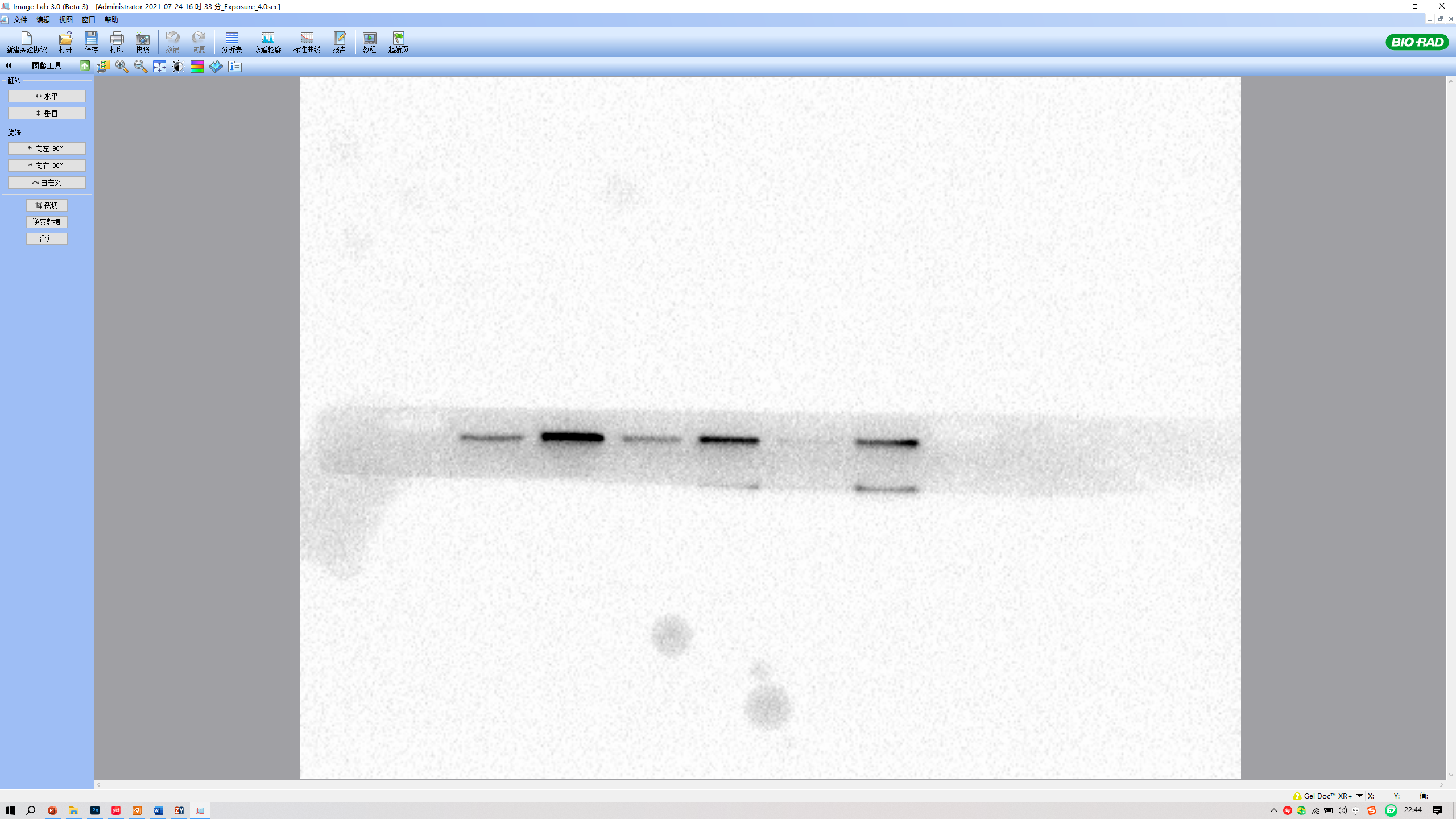


**YY1**

**GAPDH**

**YY1**

**GAPDH**

**Fig 3C**

**
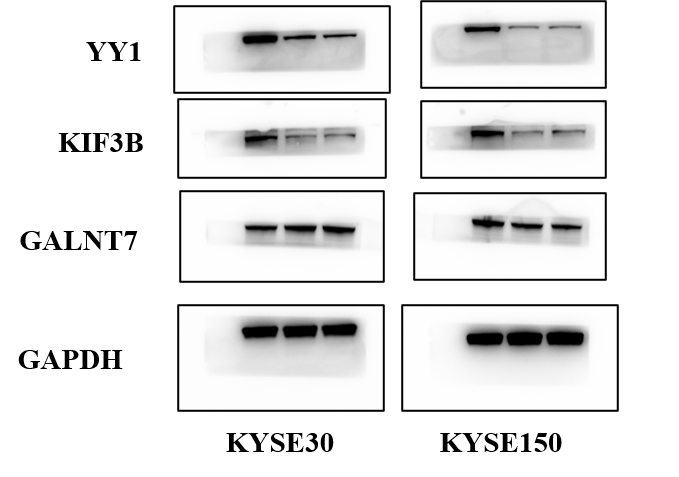
**

**Fig 4B**

**
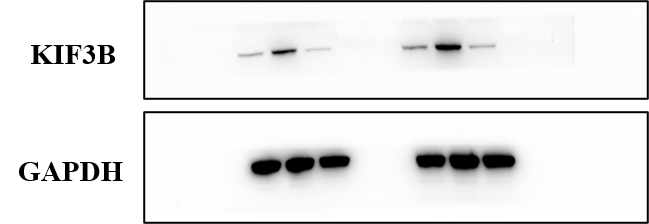
**

**Fig 5D**

**
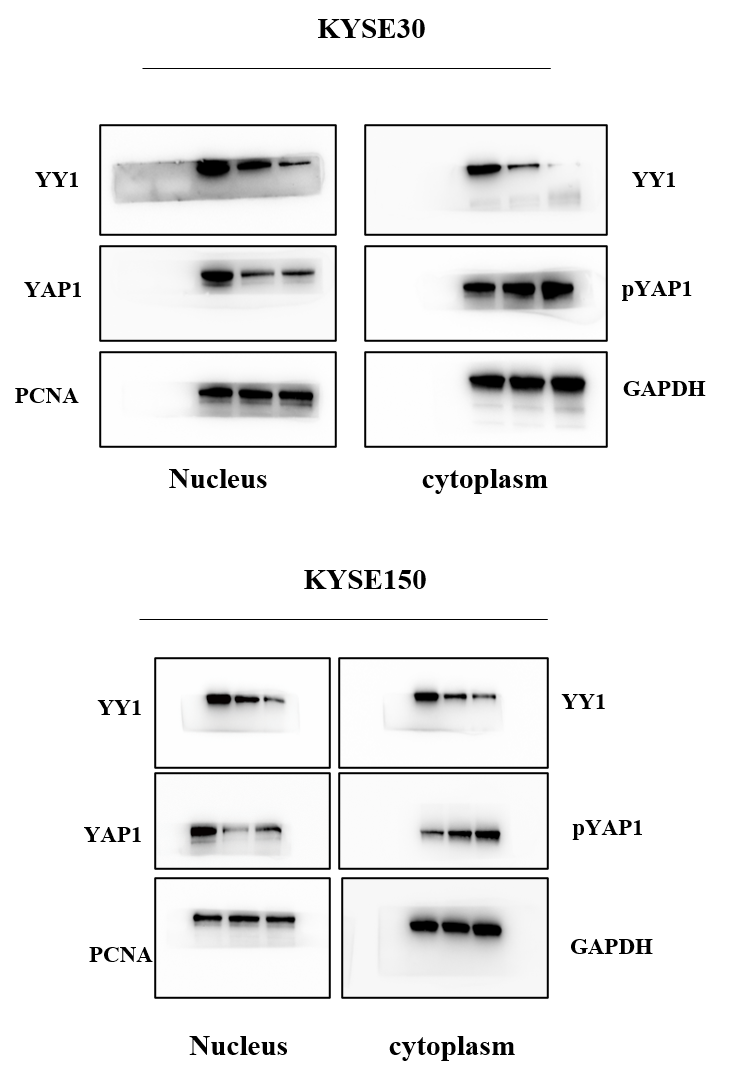
**

**Fig 5E**

**
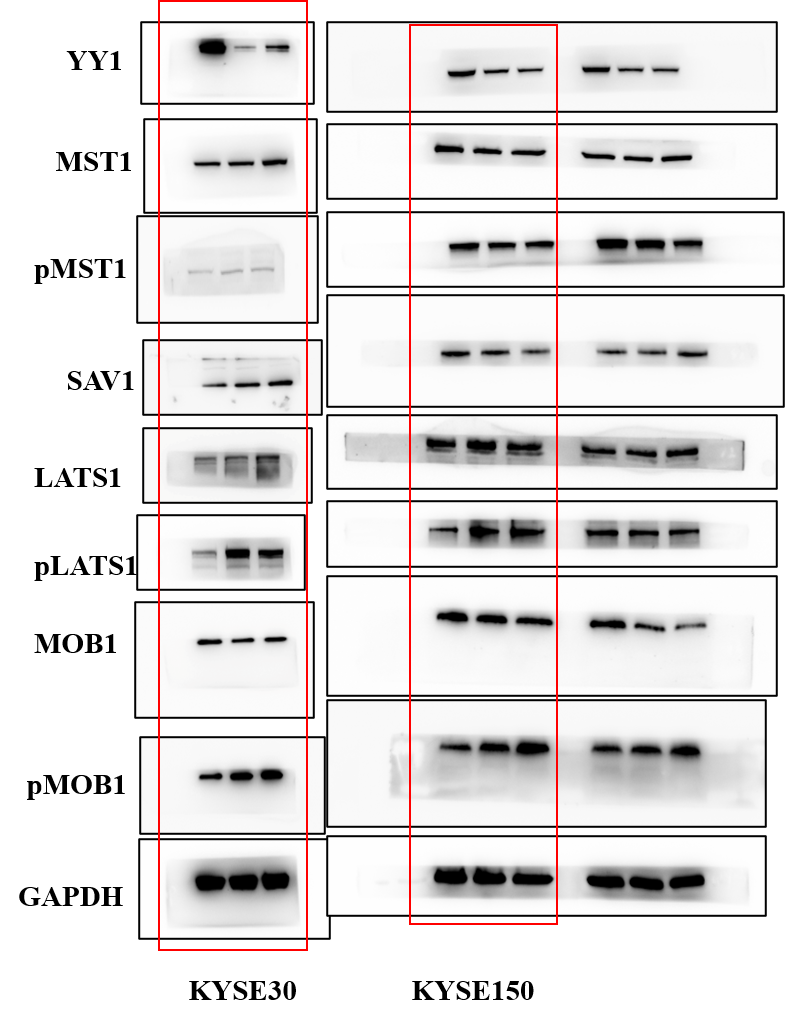
**

**Fig 5G**

**
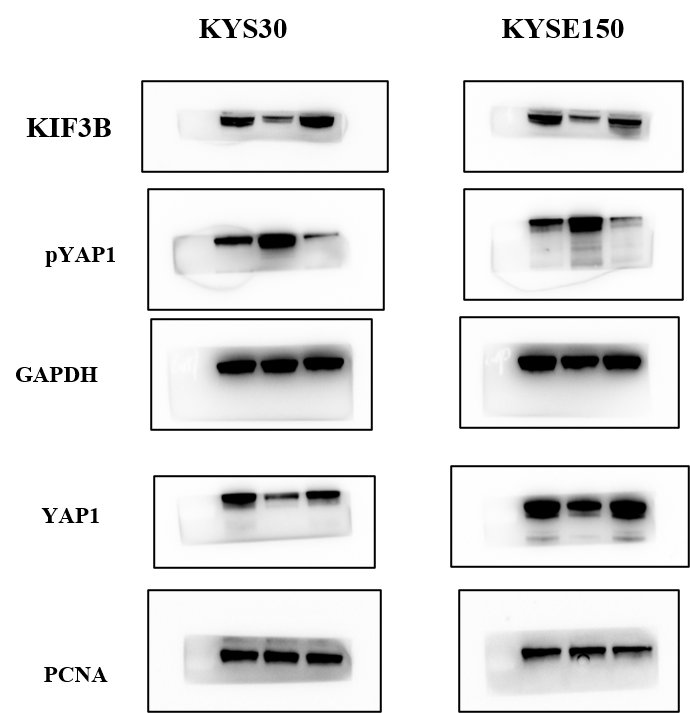
**

**Fig 5H**

**
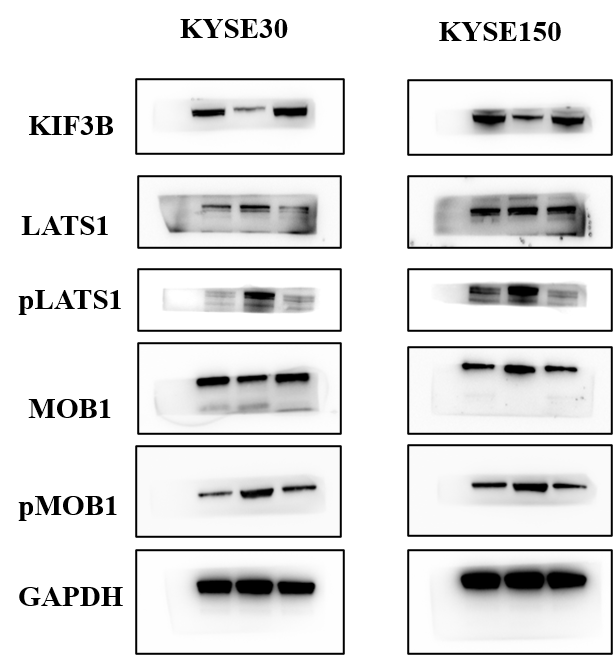
**

**Fig 5I**

**
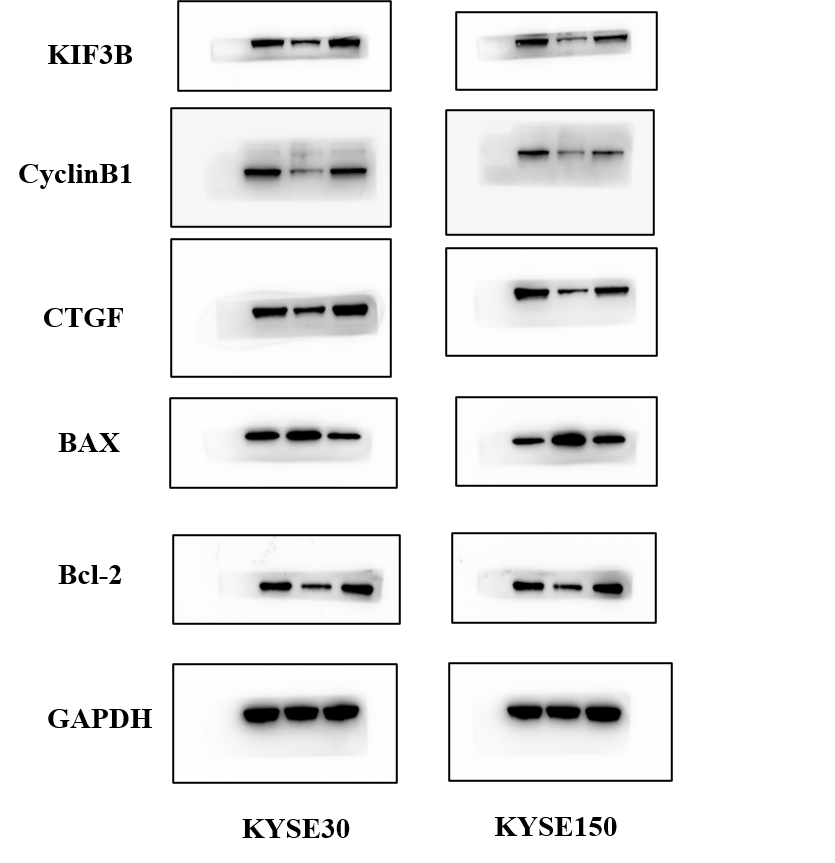
**

**Fig 6B**

**
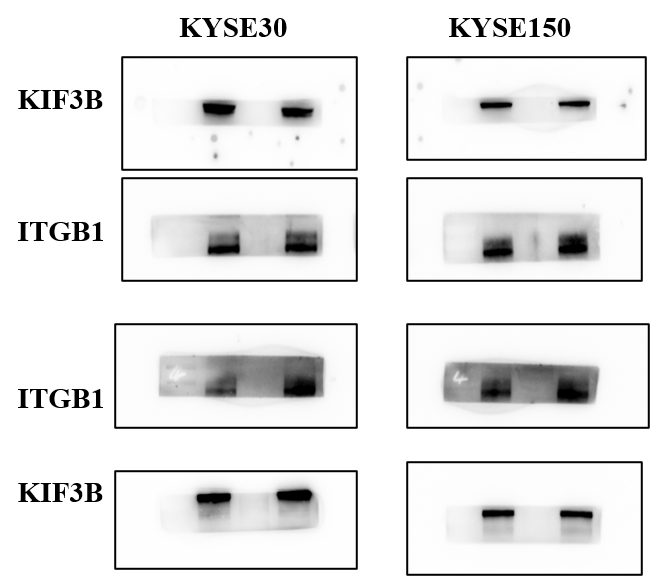
**

**Fig 6D**

**
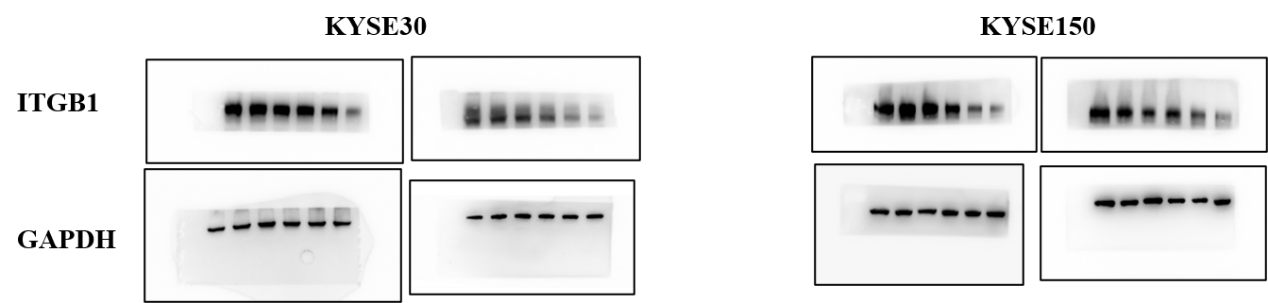
**

**Fig 6E**

**
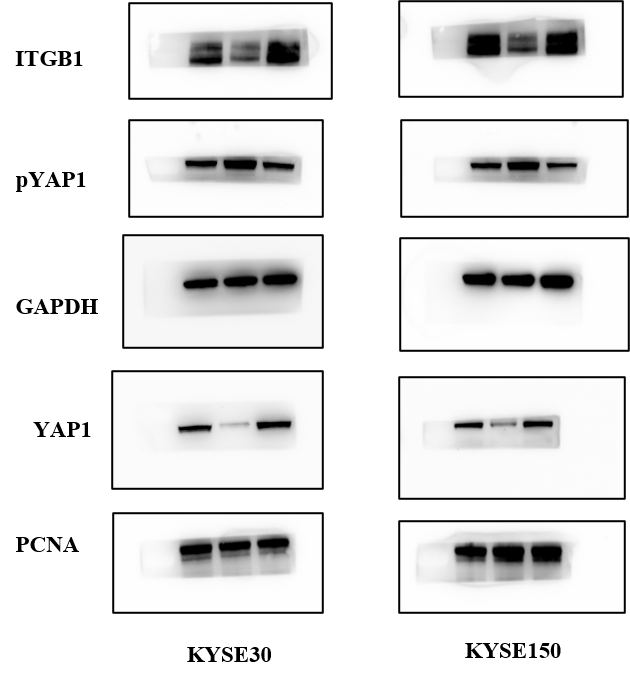
**

**Fig 6F**

**
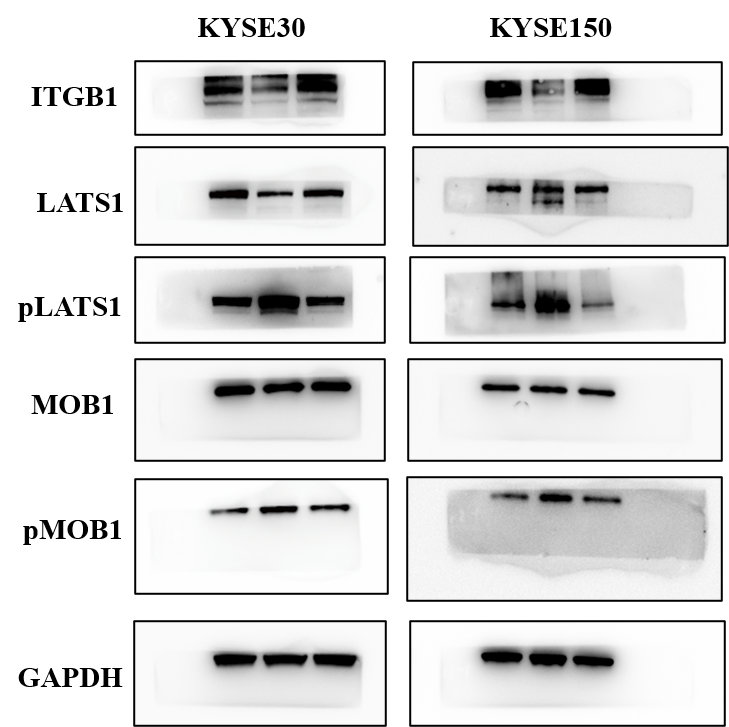
**

**Fig 7A**

**
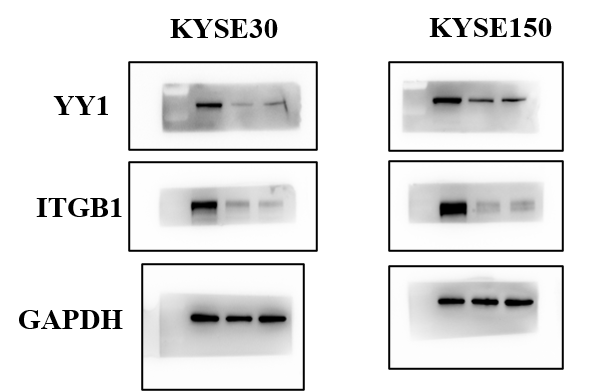
**

**Fig 7C-D**

**
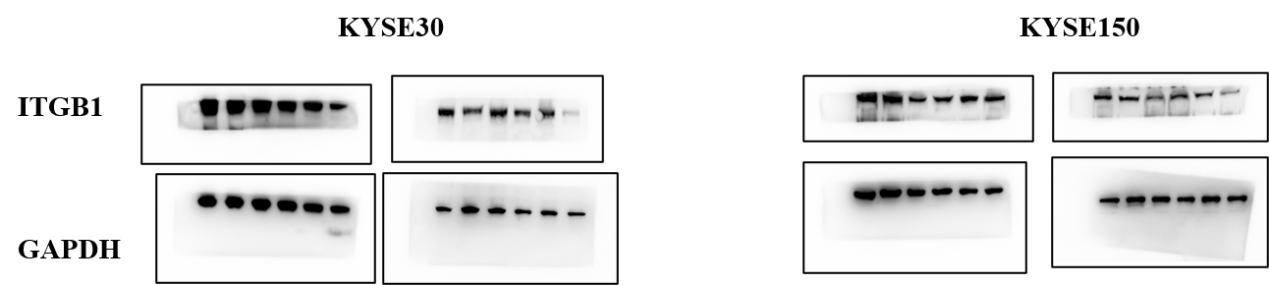
**

**Fig S3D**

**
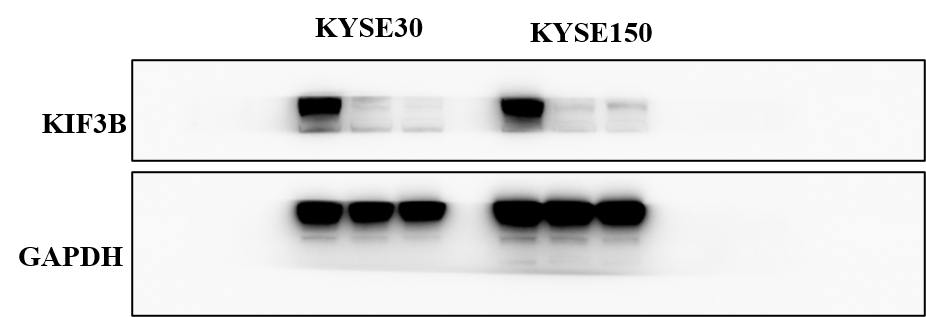
**

**Fig S6B**

**
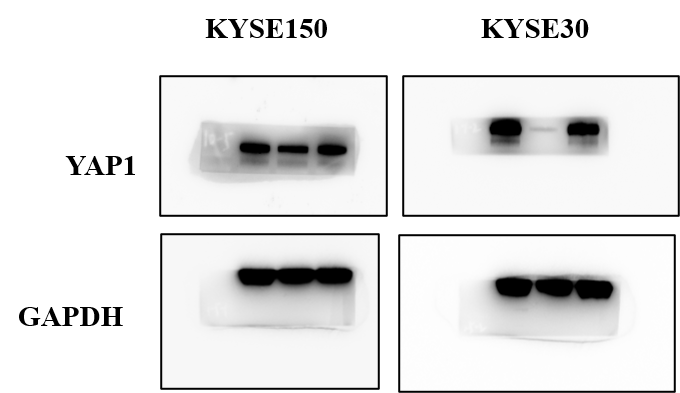
**

**Fig S7C**

**
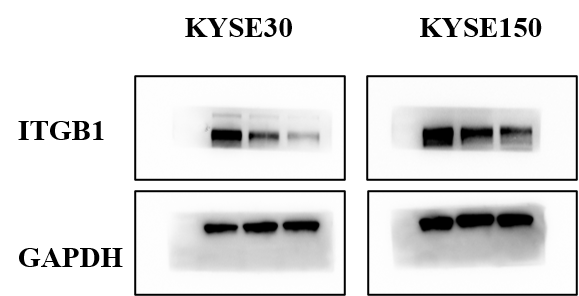
**
